# Predictive modeling of treatment resistant depression using data from STAR*D and an independent clinical study

**DOI:** 10.1371/journal.pone.0197268

**Published:** 2018-06-07

**Authors:** Zhi Nie, Srinivasan Vairavan, Vaibhav A. Narayan, Jieping Ye, Qingqin S. Li

**Affiliations:** 1 Department of Computational Medicine and Bioinformatics, University of Michigan, Ann Arbor, MI, United States of America; 2 Department of Electrical Engineering and Computer Science, University of Michigan, Ann Arbor, MI, United States of America; 3 Neuroscience Therapeutic Area, Janssen Research & Development, LLC, Pennington, NJ, United States of America; 4 Research Information Technology, Janssen Research & Development, LLC, Pennington, NJ, United States of America; Istituto Superiore Di Sanita, ITALY

## Abstract

Identification of risk factors of treatment resistance may be useful to guide treatment selection, avoid inefficient trial-and-error, and improve major depressive disorder (MDD) care. We extended the work in predictive modeling of treatment resistant depression (TRD) via partition of the data from the Sequenced Treatment Alternatives to Relieve Depression (STAR*D) cohort into a training and a testing dataset. We also included data from a small yet completely independent cohort RIS-INT-93 as an external test dataset. We used features from enrollment and level 1 treatment (up to week 2 response only) of STAR*D to explore the feature space comprehensively and applied machine learning methods to model TRD outcome at level 2. For TRD defined using QIDS-C_16_ remission criteria, multiple machine learning models were internally cross-validated in the STAR*D training dataset and externally validated in both the STAR*D testing dataset and RIS-INT-93 independent dataset with an area under the receiver operating characteristic curve (AUC) of 0.70–0.78 and 0.72–0.77, respectively. The upper bound for the AUC achievable with the full set of features could be as high as 0.78 in the STAR*D testing dataset. Model developed using top 30 features identified using feature selection technique (k-means clustering followed by *χ*^2^ test) achieved an AUC of 0.77 in the STAR*D testing dataset. In addition, the model developed using overlapping features between STAR*D and RIS-INT-93, achieved an AUC of > 0.70 in both the STAR*D testing and RIS-INT-93 datasets. Among all the features explored in STAR*D and RIS-INT-93 datasets, the most important feature was early or initial treatment response or symptom severity at week 2. These results indicate that prediction of TRD prior to undergoing a second round of antidepressant treatment could be feasible even in the absence of biomarker data.

## Introduction

Treatment resistant depression (TRD) and relapse/recurrence of major depressive episodes (MDE) are important sources of morbidity and mortality. Early Identification of risk factors of resistance using baseline characteristics and initial response may be useful to guide treatment selection, avoid inefficient trial-and-error, alter disease course, and improve major depressive disorder (MDD) care. The term initial and early response are used interchangeably in this article and its refers to response at 2 weeks.

In the literature, there are multiple TRD definitions[1] and staging models such as the Antidepressant Treatment History Form (ATHF), the Thase and Rush Model, the European Staging Model, the Massachusetts General Hospital Staging Model, the Maudsley Staging Model (MSM), [2] and the Dutch Measure for quantification of TRD (DM-TRD), an extension of MSM.[3] From the health authority’s perspective, the September 17, 2009 Committee for Medicinal Product for Human Use (CHMP) Concept Paper on “The Need For Revision of Note for Guidance on Clinical Investigation of Medicinal Products In The Treatment of Depression With Regard To Treatment Resistant Depression” stated that “In a clinical pragmatic view a patient has been considered suffering from TRD when consecutive treatment with two products of different pharmacological classes, used for a sufficient length of time at an adequate dose, fail to induce a clinically meaningful effect (inadequate response).” However, since there is no evidence supporting switching of treatment within one class is less effective than switch to a different pharmacologic class, the May 23, 2013 CHMP Guideline on “clinical investigation of medicinal products in the treatment of depression” stated that “For the purpose of this guideline TRD is considered, when treatment with at least two different antidepressant agents (of the same or a different class) prescribed in adequate dosages for adequate duration and adequate affirmation of treatment adherence showed lack of clinically meaningful improvement in the regulatory setting.” We adopted the spirit of 2013 CHMP definition in this study. A failed antidepressant trial could be defined by either failure to respond (less than 50% reduction in depression severity score) or failure to reach remission (such as having the 16-item Quick Inventory of Depressive Symptomatology, clinician-rated (QIDS-C_16_) score > 5 [4]). The risk factors for TRD as reviewed by Bennabi et al., 2014 [5] include clinical risk factors such as comorbid anxiety disorder, current suicidal risk, non-response to the first antidepressant received in the patient's lifetime and presence of melancholic features [6]; bipolarity, early onset of first depressive episode, high rate of depressive recurrences, and lack of full remission after a previous episode [7]; low reward dependence and low cooperativeness [8]; high neuroticism, low extraversion, low openness, and low conscientiousness [9]. In addition, a subset of patients with diagnosis of "unipolar" treatment resistant depression may have a bipolar diathesis[10].

Although there are many studies using baseline clinical risk factors/symptom clusters and/or early treatment response to predict longer term antidepressant treatment outcome within the same treatment regime in clinical ascertained samples [11–14], the studies using baseline clinical characteristics and initial response to earlier treatment regime to predict response to next treatment option are limited. Perlis et al. [15] developed a clinical decision tool to predict the risk of TRD based on clinical or socio-demographic variables available by or readily amenable to self-report using a logistic regression model with area under the receiver operating characteristic curve (AUC) AUC exceeding 0.71 in training, testing, and validation datasets, all derived from the Sequenced Treatment Alternatives to Relieve Depression (STAR*D). In the work by Perlis et al., non-TRD and TRD subjects were defined as individuals reaching remission with a first or second pharmacological treatment trial and those not reaching remission despite two trials, respectively. In our proposed work, we extended the work by Perlis et al., via partitioning the data from STAR*D cohort into the training and the testing dataset, and included a completely independent cohort RIS-INT-93 as an external test dataset. In addition, we explored the definition of non-TRD and TRD as individuals responding with a first or second treatment trial and those not responding despite two trials, respectively. In the work by Perlis et al., performance of three machine learning models, namely, naïve Bayes classifier, support vector machine (SVM), and random forest, was less consistent. In our work, we further explored other machine learning models such as XGBoost, *l*_2_ penalized logistic regression, gradient boosted decision tree (GBDT), the elastic net and compared their performance in the prediction of TRD.

## Methods

### Cohorts

#### STAR*D cohort

The multicenter antidepressant effectiveness STAR*D study (ClinicalTrials.gov number NCT00021528) has been described elsewhere.[16, 17] Briefly, patients meeting DSM-IV criteria for MDD went through four levels of treatment options, for up to 12 weeks in length at each level. A patient exited the study and had the option to enter a naturalistic follow up study if achieving remission at the end of each level of treatment. Otherwise the patient had the option to enter the next level of treatment. All subjects signed written informed consent before participation, with the protocol approved by institutional review boards at participating institutions.

#### RIS-INT-93 cohort

Data were drawn from a Janssen clinical study RIS-INT-93 (ClinicalTrials.gov number NCT00044681)[18]. Patients met DSM-IV criteria for MDD and had history of resistance to therapy with antidepressant medication and were treated (open-label) prospectively with citalopram for up to 6 weeks. Since the first level of antidepressant failure was retrospective, baseline (week 0) and early response at week 2 from the level 2 prospective treatment was used in TRD modeling. All subjects signed written informed consent before participation, with the protocol approved by institutional review boards at participating institutions.

### Design

#### STAR*D training and testing, and an external test dataset

Data from the STAR*D cohort was divided into the training and the testing datasets. The training dataset was drawn from ~80% of the STAR*D data from regional centers 1–8, 10–12), while the testing dataset was drawn from the rest (~20%) of the STAR*D data from regional centers 13–15 also termed as the hold-out testing dataset. Internal cross-validation was performed using the STAR*D training dataset to tune model parameters. Data from RIS-INT-93 cohort served as an independent external test dataset.

#### Target variable for predictive modeling

The predictive modeling objective is to differentiate TRD from non-TRD as early as possible. Non-TRD and TRD subjects were defined by remission as individuals reaching remission after Level one or Level two of treatment trials and those not reaching remission despite two trials, respectively; and by response as individuals responding after Level one or Level two of treatment trials and those not responding despite two trials, respectively. For the STAR*D cohort, remission was defined as a score of < = 5 on QIDS-C_16_ (primary analysis) or Quick Inventory of Depressive Symptomatology, Self-Report (QIDS-SR_**16**_, secondary analysis) at last observation carried forward (LOCF). Response was defined as a reduction of > = 50% in baseline QIDS-C_16_ or QIDS-SR_**16**_ score. For both remission and response criteria, patients must have a QIDS score greater than 5 at week 0 (baseline) of treatment level one, and remained in the study for at least four weeks. Only data from the first two levels of treatment were used in the STAR*D study.

For RIS-INT-93, remission was defined as a score of < = 7 on HAM-D_17,_ while response was defined as reduction of > = 50% in baseline HAM-D_17_ score after 6 weeks of citalopram treatment. Since all patients were retrospectively reported to have failed one round of antidepressant treatment, all non-remitters/non-responders from the prospective treatment were classified as TRD, while others were classified as non-TRD. In addition, subjects must remain in the study for at least six weeks to be included in the analysis.

#### Input features for predictive modeling

Three sets of features were included in the modeling, the full set of features (n~700) (referred to later as full set of features), the top n features from feature selection technique (referred to later as the top n features), and the set of 22 overlapping features between the STAR*D and RIS-INT-93 datasets (referred to later as overlapping features).

**Full set of features**: All the features from enrollment including information from Cumulative Illness Rating Scale (CRS), demographics (DM), psychiatric history (PHX), medication history (MHX), the Patient Rated Inventory of Side Effects (PRISE), Psychiatric Diagnostic Screening Questionnaire (PDSQ), as well as baseline and week 2 of level 1 treatment which include records from Clinic Visit Form, QIDS-C_16_, QIDS-SR_**16**_). Each attribute related to medications was expanded into as many features as number of medications that appeared in the data with each feature representing information collected on a specific mediation. Additional features were derived based on prior knowledge and included in predictive modeling. For comorbid condition, any anxiety disorder (including posttraumatic stress disorder, panic disorder with or without agoraphobia, specific phobia, social phobia, and general anxiety disorder) was derived. In addition, studies suggest that the unidimensional subscales of the multidimensional HAM-D_17_,[19, 20] which capture the core depressive symptoms, outperform the full HAM-D_17_. We included five unidimensional subscales as discussed by Boessen et al.,[21] including the Bech melancholia scale,[22–24], the Maier-Phillipp severity subscale,[25–27] the Santen Subscale,[28] the Gibbons’ global depression severity scale,[29] and the 7-item abbreviated version (HAM-D_7_).[30] The five factors derived from HAM-D_17_ (retardation (the sum of the scores for items 1, 7, 8 and 14, with a score ranging from 0 to 14), anxiety/somatization (the sum of the scores for items 10, 11, 12, 13, 15, and 17, with a score ranging from 0 to 18), sleep disturbance (the sum of scores for items 4, 5, and 6, with a total score ranging from 0 to 6), depression, guilt association) was also included as candidate features and tested if any of them was a better predictor than HAM-D_17_ item-level data or full HAMD score. Both IDS-C_30_ and HAM-D_17_ were only measured at level entry and exit, while QIDS-C_16_ and QIDS-SR_**16**_ were measured every two weeks, we derived an exploratory variable IDS-C_5_ that was definable from QIDS-C_16_ and overlapped with five out of the six items defining IDS-C_6_/HAM-D_6_[24, 31], the Bech melancholia scale. Out of the six items (mood sad, involvement, fatigue, anxious mood, outlook (self), and psychomotor slowing) defining IDS-C_6_, all except anxious mood was definable from QIDS-C_16_ or QIDS-SR_**16**_. We included percentage change of IDS-C_5_ and QIDS-C_16_ at week 2, QIDS-C_16_ at week 2 in the predictive model as well. For each data set, we eliminated all the features that have a variance less than 10^−8^ across the selected set of samples. After this preprocessing, we were left with around 700 starting features for each dataset.

**Top n features:** All machine learning approaches did not apply feature selection prior to model fitting. Many of the features were correlated with each other (such as QIDS-C_16_ was correlated with QIDS-SR_**16**_ and HAM-D_17_) and most of the machine learning approaches considered in this work do not handle correlated features well. To mitigate this and to facilitate model interpretation and application, we applied two strategies to select limited sets of features for machine learning models. The primary feature selection strategy was using a similar approach as proposed in Bühlmann et al.[32]. It is a two-step process and it as follows:

Step 1: K-means clustering was first applied to all features to group features into k groups (k = 50, 75, and 100 in our experiment). The feature closest to the centroid of each cluster was chosen as the representative feature for the cluster.

Step 2: To this end, a *χ*^2^ score as proposed by Liu et al [33] was calculated as a measure of feature importance to select top n features for model creation. Its implementation is available at http://featureselection.asu.edu/software.php. The method discretizes each numeric attribute based on *χ*^2^ statistics and continuously merges the intervals of each attribute while keeping the inconsistency rate under certain threshold. We adopted this method due to the ordinal nature of majority of the features such as depression symptom severity score as measured by QIDS-C_16_ and HAM-D_17_ and those measuring how depression affects patients’ daily life as exemplified by items from Work and Social Adjustment Scale (WSAS) which gives a quantitative evaluation of to what extent depression impairs activities related to social and work life. The *χ*^2^ statistics was calculated using the STAR*D training dataset or the 90% of the training dataset (during 10-fold cross-validation procedure within the training data set for optimization of model parameters). This procedure was applied for each of the target outcome and for each feature clustering configuration (k = 50, 75, 100). This feature selection strategy is called clustering- χ^2^ throughout the article.

The second feature selection strategy was using the elastic net technique [34, 35] and it corresponds to n features with non-zero beta coefficient while optimizing the number of non-zero beta coefficient to ~30. This feature selection strategy is termed as ELNET features throughout the article. As optimizing elastic net to approximately 30 features could result in sub-optimal performance, we selected top n features optimized according to the model’s performance (area under the receiver operating characteristics curve (AUC)) via 10-fold internal cross-validation in the training dataset and the results were comparable. If not explicitly stated, the ELNET features refers to the ~30 features selected by elastic net.

**Overlapping features**: There were 22 overlapping (but not exhaustive list of) features between STAR*D and RIS-INT-93 (week 0 17 HAMD item score, week 0 HAM-D_17_ total score, HAM-D retardation subtotal score, percentage change of HAM-D_17_ total score at week 2 from baseline, any anxiety disorders, and recurrent depression) and represented the third set of features for the prediction of TRD.

### Predictive modeling

#### Machine learning methods

Five different machine learning approaches including *l*_2_ penalized logistic regression [36, 37] random forest [38] GBDT, XGBoost, and the elastic net were applied in this study. The implementations of *l*_2_ penalized logistic regression, random forest, GBDT, the elastic net available from scikit-learn [39] and XGBoost from https://xgboost.readthedocs.org/en/latest/ were used in this study.

#### Handling class imbalance

**Fig 1:**
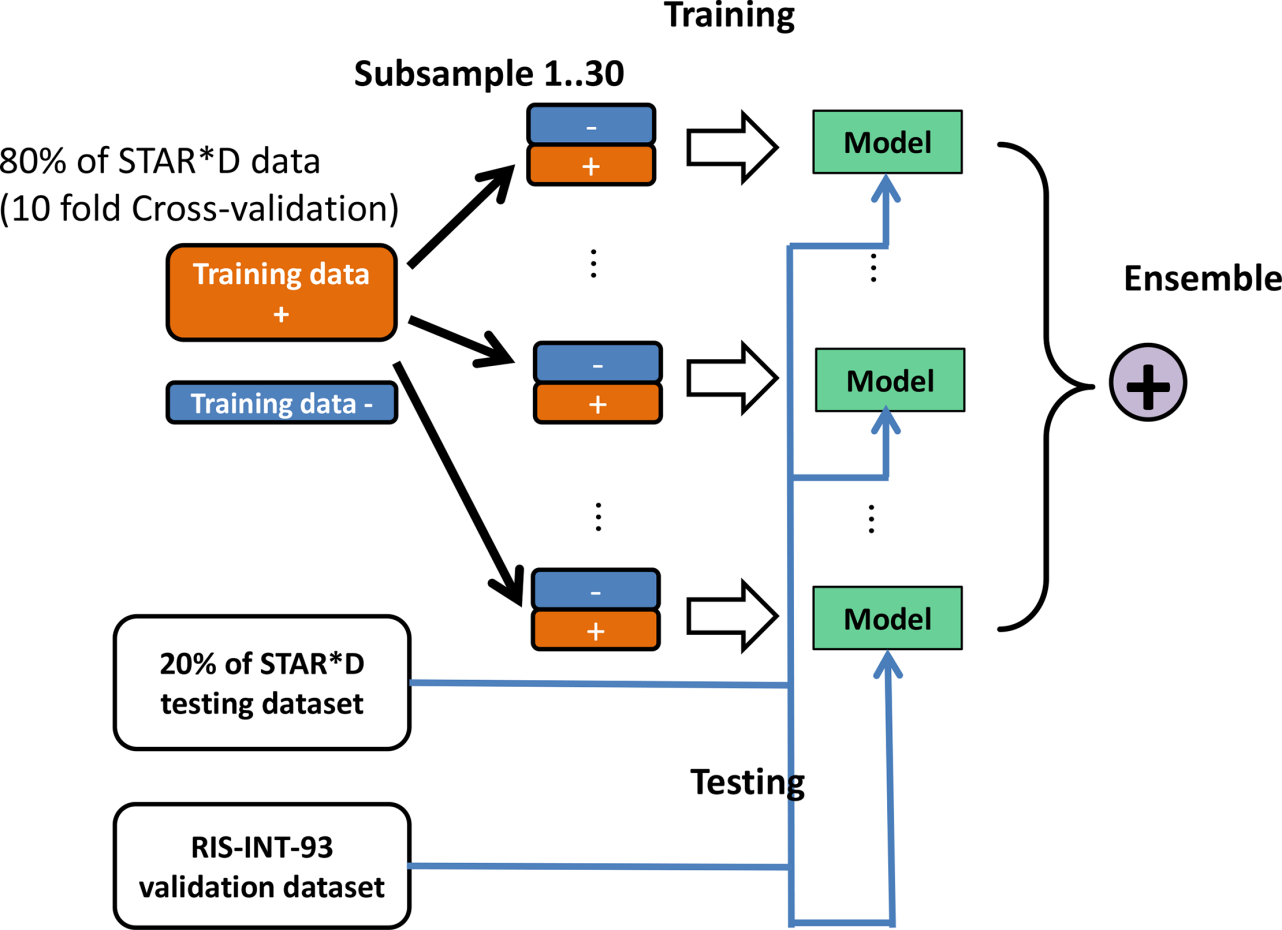
Schematic diagram showing the predictive modeling process. The STAR*D training dataset was used to create 30 subsamples with equal ratios of cases and controls and 30 models were constructed using the entire training dataset. The 30 models were used to predict the outcome for the independent STAR*D test dataset and RIS-INT-93 dataset and the predicted outcome were average across 30 models.

In our prediction tasks, subjects from different categories (remission vs. non-remission; response vs. non-response as determined by either QIDS-C_16_ or QIDS-SR_16_) were imbalanced to different degrees. A direct application of the machine learning model introduced above would lead to the obtained model biased towards the majority class. The class imbalance handling technique that has been used in Nie et al., 2015 [40] was also applied to the training data in STAR*D cohort. Basically, during the training stage, we used all the samples from minority class in the training dataset and subsample with replacement an equal number of samples from the majority class to build one model. We repeated the process *t* times and thus obtained *t* models (*t* = 30). In obtaining predictions for the test dataset, an ensemble model prediction corresponding to *t* models are obtained by averaging *t* model predictions (probabilities) and the performance are assessed against the target. (Fig 1).

#### Model characterization

Internal cross-validation (10-fold) performance metrics was reported for the training dataset using full set of features and top n features (n = 2, 3, …, k, where k = 50, 75, and 100 clusters). The model derived from the STAR*D training dataset was externally tested in a hold-out STAR*D testing dataset. For the overlapping features, the models trained using the STAR*D training dataset was both internally cross-validated in the training dataset and externally validated in the hold-out STAR*D testing data and an independent RIS-INT-93 dataset using machine learning approaches. Relevant descriptions of model performance including AUC, sensitivity, specificity, accuracy, balanced accuracy, positive predictive value (PPV), and negative predictive value (NPV) were determined.

#### Permutation test

To make sure that the observed model performance is not obtained by chance, the target label was randomly permutated 1,000 times in the START*D training dataset. The model’s training on the STAR*D training dataset with permuted target labels and the prediction process in STAR*D testing dataset was repeated for 1,000 times giving rise to a null distribution of AUC values. The observed AUC value was plotted in reference to the null AUC distribution, and a permutation p-value was calculated. [41]

#### Interpretation of the relationship between features and outcome via multiple logistic regression procedure

Classical logistic regression with forward selection was used to facilitate model interpretation, although only linear relationship could be captured in the model. With this approach, only the top n features from the STAR*D training data (n = 30 from clustering-χ^2^) were included in the model as candidates for forward selection. The features selected were included in a multiple logistic regression model and features with p-value less than 0.1 were examined to understand the relationship between features and outcome. Classical logistic regression was fitted using PROC LOGISTIC via SAS 9.2 (SAS Institute Inc., Cary, NC).

## Results

Using the remission criteria, there were 501 TRD cases and 1463 non-TRD controls from the STAR*D training dataset and 141 TRD cases and 349 non-TRD controls from the STAR*D testing dataset using QIDS-C_16_ assessment (primary analysis, S1 Table). For RIS-INT-93, there were 200 TRD cases and 25 non-TRD controls definable using HAM-D_17_. Using the responder criteria, there were 411 TRD cases and 1797 non-TRD controls from the STAR*D training dataset and 104 TRD cases and 470 non-TRD controls from the STAR*D testing dataset using QIDS-C_16_ assessment. For RIS-INT-93, there were 190 TRD cases and 35 non-TRD controls definable using HAM-D_17_.

**Fig 2:**
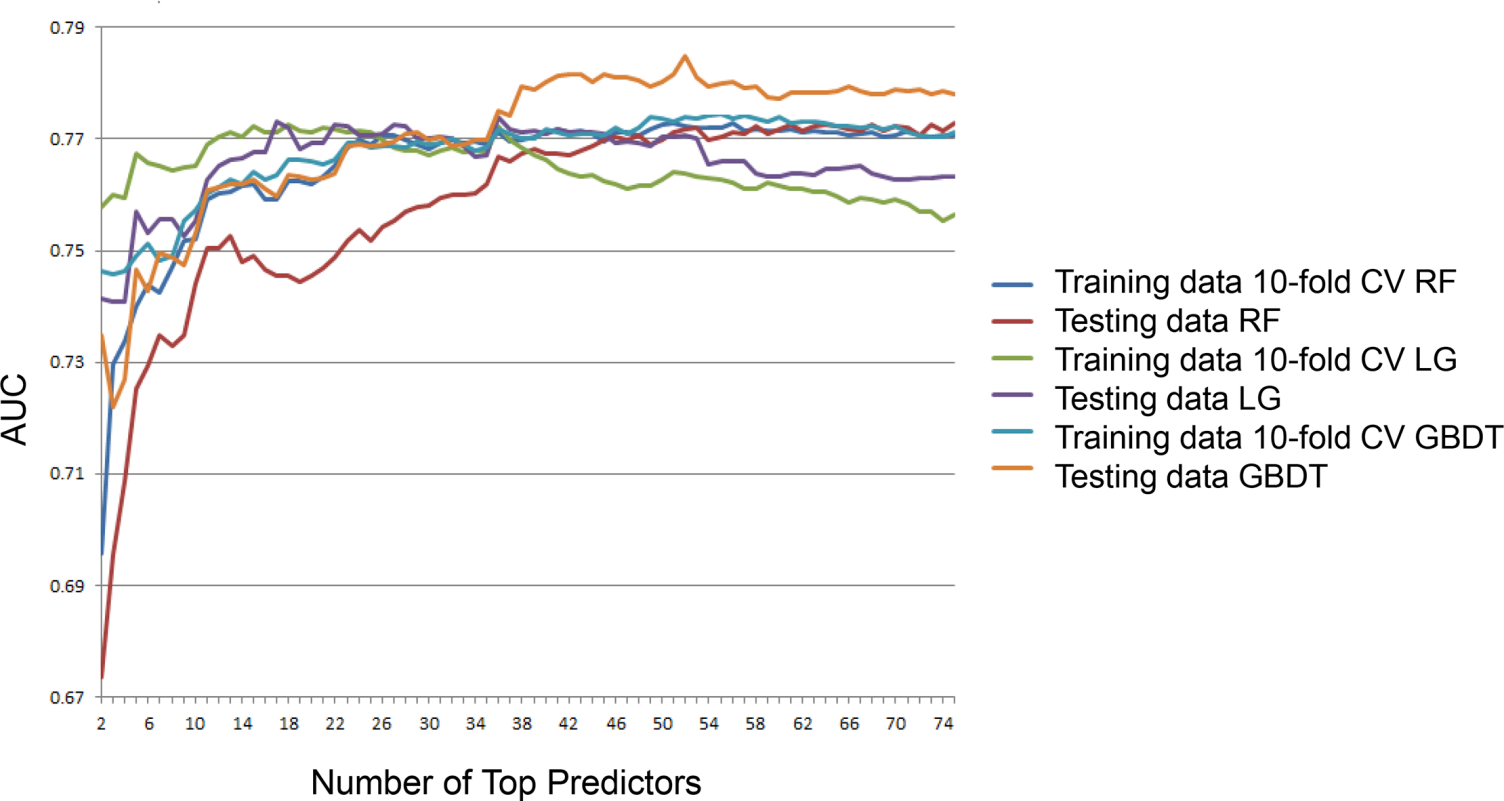
AUC for models containing top 2 to 75 representative predictors from k-means cluster (k = 75) was plotted against the number of predictors for each of the machine learning methods in the STAR*D training and test datasets, respectively. Remission status was used to define TRD using QIDS-C_16_ data.

To reduce the correlation between features, we used k-means clustering to group features into clusters and select a candidate feature representing each cluster. To explore the approximate number of top features that should be included in the predictive model without compromising on the model performance, the top n features (n = 2, 3, …, 75) from k-means clustering (k = 75 for k-means clustering, as results from k = 50 and 100 were comparable) were used to train, test the model (as shown in Fig 2) and the model’s performance plateaued at ~10–30 predictors. The variance of AUC obtained from internal cross-validation in the STAR*D training dataset for each fold was averaged across 30 models corresponding to 30 subsamples. The variance for the top n (n = 30) features was 3.9%, 3.3%, and 3.3% for random forest, *l*_2_ penalized logistic regression, and GBDT, respectively. The results for full set of features and the top n features (n = 30) were reported for subsequent analysis. We also used the ELNET features generated by elastic net (optimizing for number of features = ~30) and compared the results to the earlier top n features. In addition, the results for the overlapping features was also reported.

**Fig 3:**
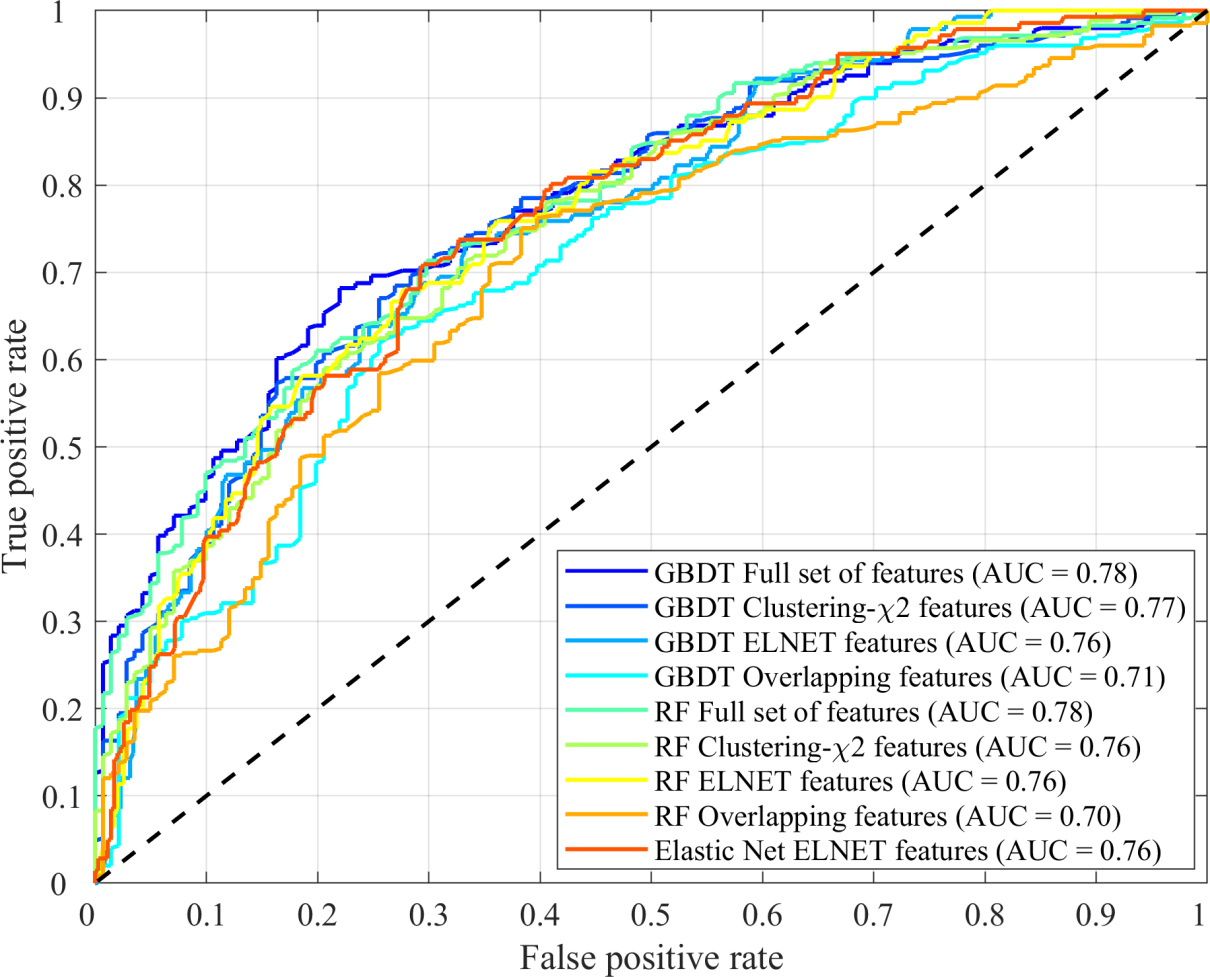
Receiver operating characteristic curves in training and test dataset (STAR*D) using the full set of features, top n features (n ~ 30), and the overlapping features where remission status was used to define TRD (STAR*D remission status was defined using QIDS-C_16_ data, and RIS-INT-93 remission status was defined using HAM-D_17_).

**Fig 4:**
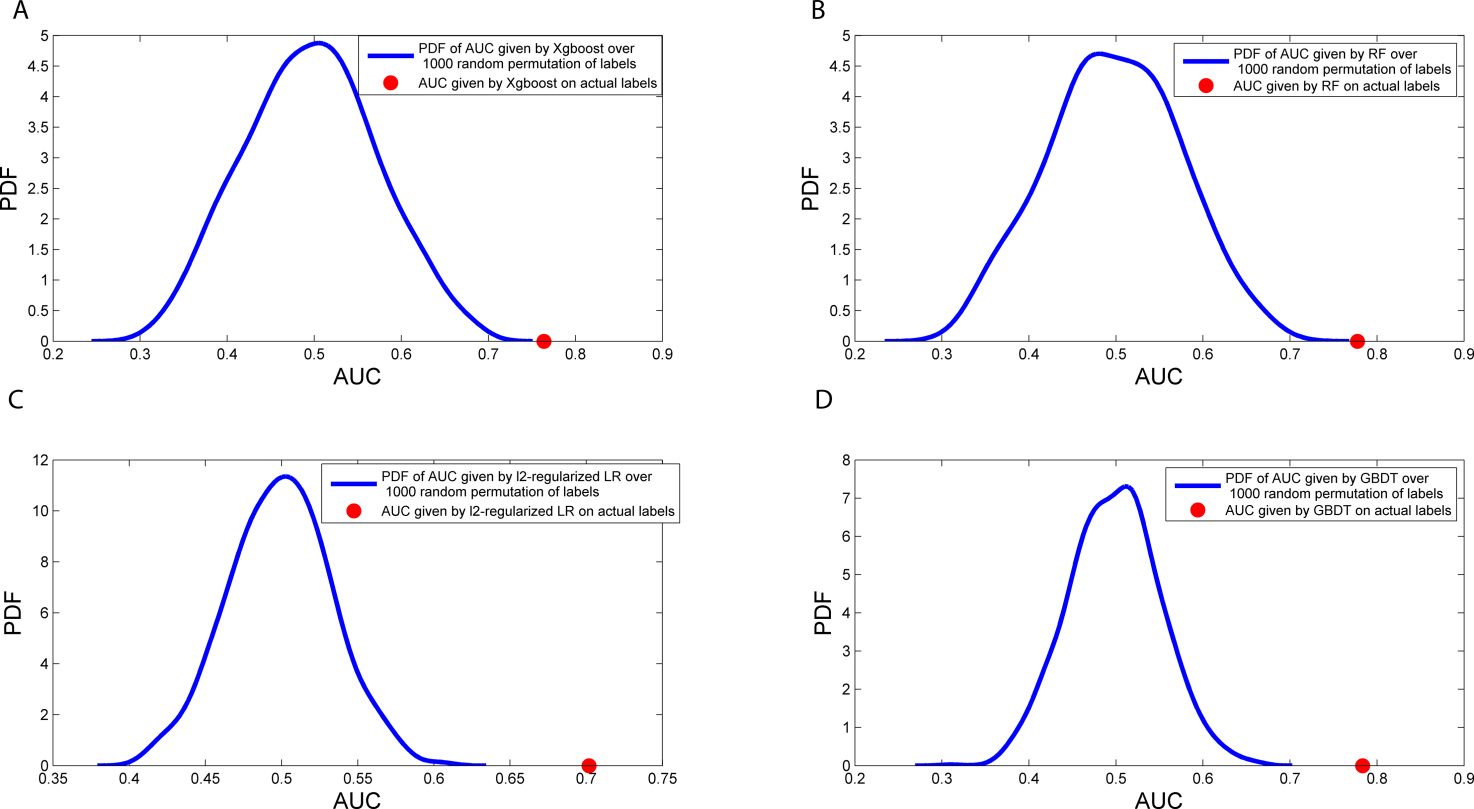
Permutation process to access the model robustness. The outcome label of the STAR*D training dataset was randomly shuffled 1,000 times and the AUC distribution of the 1,000 null models were plotted for each machine learning machine method (A) XGBoost, (B) Random Forest, (C) *l*_2_ penalized logistic regression, and (D) GBDT. In all cases, the observed AUC out-perform the random noise from the 1,000 null models.

For TRD phenotype defined by remission criteria using QIDS-C_16_ assessment, the AUC for the 10-fold internal cross-validation in the training dataset ranged from 0.73 to 0.79 with the full set of features with GBDT performing the best (S1A Fig). The out of bag (OOB) score for random forest was 0.71. The model derived from the training data was externally validated in the STAR*D test dataset with the AUC ranging from 0.70 to 0.78 for all different machine learning approaches and is shown in Fig 3 and S1D Fig. The permutation testing for all the machine learning approaches is shown in Fig 4. Model AUC, accuracy, specificity, sensitivity, PPV, and NPV for selected models were also reported in Table 1, S2 Table, and S3 Table using predicted target (ranging from -1 to 1) with 0 as a threshold for classifying predicted target. The full set of features represented the upper bound of model performance using all clinical variables. To remove the correlated structure among the features, the AUCs for the top n features (n = 30) (from the k-means clustering procedure were 0.76–0.77 in the STAR*D training data (S1B Fig) and 0.75–0.77 in the STAR*D testing data (S1E Fig). To test if the model could be generalized to a completely independent dataset, namely, RIS-INT-93, the overlapping features were both internally cross-validated in the STAR*D training data (10-fold cross-validation AUC 0.70–0.71, S1C Fig) and externally validated in the STAR*D hold out testing data (AUC 0.70–0.73, S1F Fig) and the independent RIS-INT-93 dataset (AUC 0.72–0.77, S1G Fig). Confusion matrix for GBDT model in STAR*D test dataset and independent dataset RIS-INT-93 was also reported in S4 Table. Similar results were observed for target variable using responder criteria defined using QIDS-C_16_ assessment (S2 Fig) and for target variable using remission and responder criteria defined QIDS-SR_16_ assessment (S3 Fig and S4 Fig). Overall, the models achieved AUC > = 0.7 in at least one machine learning approaches across the STAR*D testing dataset and RIS-INT-93 dataset and was independent of the phenotype definition by remission or response criteria or using QIDC-C_16_ or QIDS-SR_16_ assessment, although the performance of TRD using remission criteria generally was better than that using response criteria and the performance of the full set of features and the top n features outperformed that of the overlapping features as expected. For simplicity of reporting, we reported models with TRD defined using remission criteria and QIDS-C_16_ assessment moving forward if not explicitly stated. Results from alternative models could be found in the supplemental content. The results for the ELNET features (elastic net optimized for number of features = ~30 for ease of comparison with the top n features selected by clustering-χ^2^) were comparable and described in S5 Table.

**Table 1:** Model performance (outcome defined by remission using QIDS-C_16_) in the STAR*D testing dataset and RIS-INT-93.

Model	Accuracy	Balanced Accuracy	Sensitivity	Specificity	PPV	NPV	AUC
STAR*D test data set							
***Random Forest***							
*Full set of features*	0.70	0.70	0.69	0.71	0.49	0.85	0.78
*Top n features (n = 30)*^*a*^	0.67	0.68	0.72	0.65	0.45	0.85	0.77
*Top n features (n = 31)*^*b*^	0.70	0.69	0.68	0.71	0.48	0.85	0.76
*Overlapping features*	0.65	0.66	0.67	0.64	0.43	0.83	0.71
***GBDT***							
*Full set of features*	0.70	0.70	0.69	0.71	0.49	0.85	0.78
*Top n features (n = 30)*^*a*^	0.70	0.70	0.72	0.68	0.48	0.86	0.77
*Top n features (n = 31)*^*b*^	0.70	0.70	0.68	0.71	0.49	0.85	0.76
*Overlapping features*	0.66	0.67	0.70	0.64	0.44	0.84	0.71
***XGBoost***							
*Full set of features*	0.67	0.68	0.72	0.64	0.45	0.85	0.76
*Top n features (n = 30)*^*a*^	0.66	0.67	0.67	0.66	0.44	0.83	0.73
*Top n features (n = 31)*^*b*^	0.67	0.68	0.72	0.65	0.45	0.85	0.76
*Overlapping features*	0.68	0.68	0.67	0.69	0.47	0.84	0.72
*l*_2_ ***penalized logistic regression***							
*Full set of features*	0.63	0.64	0.65	0.62	0.41	0.81	0.69
*Top n features (n = 30)*^*a*^	0.71	0.71	0.68	0.73	0.50	0.85	0.73
*Top n features (n = 31)*^*b*^	0.72	0.71	0.68	0.73	0.51	0.85	0.77
*Overlapping features*	0.67	0.67	0.68	0.66	0.45	0.84	0.74
***Elastic net***							
*Top n features (n = 31)*^*b*^	0.70	0.68	0.64	0.73	0.49	0.83	0.76
***RIS-INT-93 dataset (Overlapping features)***							
*Random Forest*	0.86	0.65	0.92	0.36	0.92	0.39	0.73
*GBDT*	0.82	0.64	0.88	0.40	0.92	0.29	0.71
*XGBoost*	0.84	0.67	0.89	0.44	0.93	0.33	0.73
*l*_2_ *penalized logistic regression*	0.83	0.64	0.88	0.40	0.92	0.29	0.60

GBDT: gradient boosting decision tree

^a^ clustering- χ^2^

^b^ELNET features

**Fig 5:**
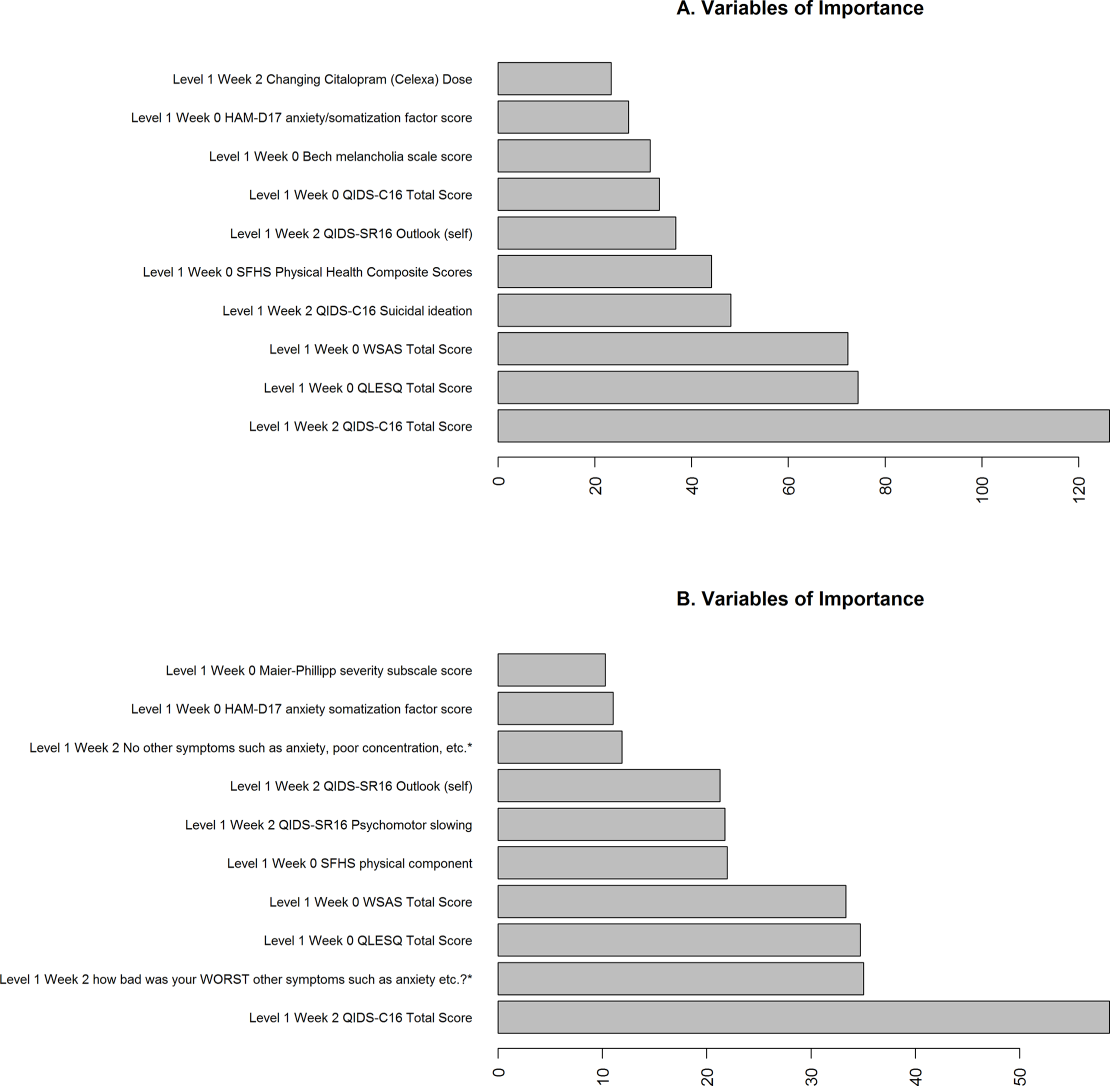
variable of importance in statistical learning approaches for outcomes defined by (A) remission status (B) responder status. In both cases, the outcomes were defined using QIDS-C_16_. SFHS: Short Form Health Survey (SF-12); WSAS: The Work and Social Adjustment Scale; *from PRISE: The Patient Rated Inventory of Side Effects, which collected symptoms one had experienced in the past week. Those symptoms may or may not have been caused by the treatment.

The top groups of features identified to be important based on the clustering- χ^2^ score and most consistent between outcome definitions (remission and response) and symptom severity measurements (QIDS-C_16_ and QIDS-SR_16_) were represented by: QIDS total score at week 2 (self-reported and clinician-rated QIDS total score were strongly correlated and grouped together with percentage change in symptom severity score at week 2 from week 0 into the same cluster by k-means clustering procedure, see S6 Table for top 20 groups of features clustered using k-means clustering), Quality of Life Enjoyment and Satisfaction Questionnaire (QLESQ) total score, Work and Social Adjustment Scale (WSAS) total score, and Short Form Health Survey (SFHS, SF-12) Physical component. The top n (n = 10) representative features from clustering- χ^2^ were also shown in Fig 5 and S5 Fig. They represented the feature importance in a univariate sense. The ELNET features (S7 Table) and were largely consistent with the univariate features of importance except that multiple correlated features were selected as expected.

To facilitate the interpretation of the relationship between features and target and to consider multiple features simultaneously (in contrast to χ^2^ statistics), both classical logistic regression model with forward selection and the elastic net and were evaluated. For the classical logistic regression, starting from top 30 features from the STAR*D training data, 8 variables were selected in forward selection procedure and 6 made it to the final logistic regression model (Table 2, S7 Table). In the STAR*D training dataset, more severe QIDS-C_16_ symptom severity total score at week 2 [or higher percentage change in QIDS-C_16_ at week 2 from baseline (i.e. less improvement) since both variables were clustered together in k-means clustering procedure (S6 Table) although QIDS-C_16_ symptom severity total score at week 0 will add additional explanatory value if including percentage change at week 2 from week 0 in the model], anxiety/chronicity (PDSQ questions), lower level of Physical Health Composite Scores (PCS), nervous system symptom (PRISE question) and WSAS total score were associated with increased odds of being TRD (Table 2). For PCS (ranging from 0 to 100), the odds ratio is less than 1 since a zero score indicates the lowest level of health measured by the scales and 100 indicates the highest level of health. Similar results for TRD defined using responder criteria were reported in S8 Table. When testing these six variables in the STAR*D testing dataset, all variables predicted outcome in the same direction as the STAR*D training data and both QIDS-C_16_ symptom severity total score at week 2 and Physical Health Composite Scores (PCS) reached statistical significance (p-value < 0.05). For the elastic net, the sign of β coefficient (S8 Table) was consistent with the directionality of odds ratio and most variables with non-zero β coefficients were consistent except correlated variables were also grouped in the model.

**Table 2:** Predictors from PROC LOGISTIC for TRD phenotype defined using remitter criteria in the STAR*D training and testing datasets.

Effect	Dataset	Pr > ChiSq	Odds Ratio Point Estimate	Odds Ratio 95% Wald Confidence Limits
QIDS-C_16_ total score at week 2 (Level 1)	Training*	< .0001	1.198	(1.161, 1.236)
	Testing*	< .0001	1.211	(1.133, 1.294)
SFHS Physical Health Composite Scores	Training	< .0001	0.978	(0.969, 0.988)
	Testing	0.0072	0.972	(0.952, 0.992)
WSAS total score	Training	< .0001	1.031	(1.016, 1.046)
	Testing	0.1072	1.023	(0.995, 1.051)
PDSQ: being in crowded places make one feel fearful, anxious, or nervous because you were afraid you'd have an anxiety attack in the situation during the past six months	Training	0.0064	1.383	(1.095, 1.747)
	Testing	0.0615	1.567	(0.979, 2.508)
PRISE: how bad was the worst nervous system symptoms (headache, tremors, poor coordination, dizziness) that one has experienced during the past week regardless of cause (0—not present, 1- tolerable, 2- distressing)	Training	0.0336	1.193	(1.014, 1.403)
	Testing	0.1813	1.270	(0.895, 1.802)
PDSQ: did you feel sad or down on most days of the past 2 years?	Training	0.0609	1.261	(0.989, 1.608)
	Testing	0.1653	1.426	(0.864, 2.354)

*Sample size for final multiple logistic regression model is 501 TRD cases vs. 1463 non-TRD controls in the STAR*D training data, and 141 TRD and 349 non-TRD in the STAR*D testing data. SFHS: Short Form Health Survey (SF-12). WSAS: The Work and Social Adjustment Scale. PRISE: The Patient Rated Inventory of Side Effects. PDSQ: Psychiatric Diagnostic Screening Questionnaire™

Lastly, we considered an overly simplified model using early response (defined by having less than 20% improvement at week 2) to predict TRD outcome. In the STAR*D training dataset, the model accuracy for predicting TRD is 62 in the STAR*D training data (sensitivity = 0.52, specificity = 0.77, PPV = 0.75, NPV = 0.54). Model performances in the STAR*D testing dataset and RIS-INT-93 dataset were comparable and is shown in S9 Table.

## Discussion

We developed a series of machine learning models to predict TRD after two trials of treatment regimens using clinical and socio-demographic data. The models were trained using three sets of features, namely, all set of features (using all available STAR*D features from enrollment and week 0 (all features) and week 2 (only symptom severity score and percentage change from baseline were used) of level 1 treatment), top n features (a subset of top 30 representative features selected by clustering- χ^2^ and elastic net), and overlapping features (a subset of 22 overlapping features between STAR*D and RIS-INT-93) were externally validated and performed reasonably well with AUC in the range from 0.70 to-0.78 in the STAR*D hold-out testing dataset and 0.74–0.78 in RIS-INT-93 independent dataset. Although the full set of features provided an upper bound using clinical and socio-demographic features, the large number of features makes it impractical to deploy the model in a real-world setting. A reduced set of top n (n = 30) features from clustering- χ^2^ procedure is more practical if we were to design a new study and collect features to predict TRD. In order to use data from existing studies as independent testing dataset, we could only work with the limitation of overlapping features.

The overall accuracy of the GBDT model using top 30 features (k = 75) was 0.70 for the STAR*D testing dataset (predicted outcome ranging from -1 to 1, using mid-point 0 as the threshold to infer TRD vs. non-TRD status). The accuracy in predicting TRD and non-TRD were comparable (0.72 and 0.68, respectively), Assuming there may be a population of ambiguous subjects who may be difficult to predict based on clinical and socio-demographic features alone, we focused only on extreme 10 and 20 percentiles of the predicted outcome distribution (of being TRD or being non-TRD). The accuracy for extreme 10 and 20 percentiles for inferring non-TRD was 0.98 (1 error out of 49 subjects) and 0.95 (5 errors out of 98 subjects), while the accuracy for extreme 10 and 20 percentiles for inferring TRD was 0.69% (15 errors out of 49 subjects) and 0.60 (39 errors out of 98 subjects), respectively, suggesting it is easier to predict non-TRD than TRD in the STAR*D testing dataset for those especially among those with extreme predicted outcome. The default choice of using mid-point in predicted probability to classify subjects is arbitrary and not necessarily the optimal threshold. We prefer to report AUC as it takes into different threshold cut points into consideration. For a predictive model to be useful in predicting individual outcome, a pre-specified threshold shall be used. We therefore also explored a specific cut point such as having a predicted score less than -0.6 predicts TRD, while having a predicted score great than 0.6 predicts non-TRD. Using these thresholds, the accuracy for non-TRD and TRD prediction was 0.90 and 0.53, respectively, in the STAR*D testing dataset. For the model using overlapping features, the overall accuracy of the GBDT model was 0.82 for RIS-INT-93 dataset. The accuracy in predicting TRD and non-TRD were 0.88 and 0.40, respectively. Using the same -0.6 and 0.6 for predicting TRD and non-TRD, the accuracy for TRD and non-TRD prediction was 0.93 (11 errors out of 154 predictions) and 0.40 (15 errors out of 25 predictions), respectively, in the RIS-INT-93 dataset. It is unlikely that the model performance and these observations merely reflect the group make up as STAR*D samples had approximately 2.5 times more non-TRD subjects than TRD subjects, while RIS-INT-93 study had more TRD than non-TRD subjects, as the actual model out-performed the null models in all cases of the permutation testing.

It is somewhat surprising that the performance of models in the independent RIS-INT-93 dataset was generally somewhat superior to the STAR*D testing dataset. In the STAR*D training dataset, symptom severity score at week 2 was selected over symptom severity score at week 0 and percentage change in symptom score, suggesting the closer the symptom measurement is to the future outcome the more predictive the feature is in predicting the future outcome. In RIS-INT-93, level 1 failure was retrospectively reported and therefore only level 2 measurements were available. Therefore, it is possible that RIS-INT-93 prediction problem might be an easier test case compared to STAR*D study design. Furthermore, the outcome measurement was at week 12 (if there is no missing data or last observation carried forward (LOCF) but minimally week 4) in STAR*D, while in RIS-INT-93 the outcome measurement was at week 6 (or earlier but at minimal week 4). In addition, the fewer number of non-TRD subjects in RIS-INT-93 (25 to 35) as against STAR*D (349 to 470) could also be a potential limitation of the current work. It is therefore important to further validate the models developed from the STAR*D training to independent studies of different design but preferably two stages of prospective interventional studies such as the Establishing Moderators and Biosignatures of Antidepressant Response in Clinical Care (EMBARC)[42–45] and the Canadian Biomarker Integration Network in Depression (CAN-BIND)[46].

## Supporting information

S1 Fig Area under the receiver operating characteristic curves (ROC AUC) for TRD as defined by remission status and QIDS-C_16_. ROC AUC in the training and test dataset (STAR*D) using the full set of features, top n features and the overlapping features in all three datasets. (A-C STAR*D training data; D-F STAR*D test data; and G RIS-INT-93 test data) where **remission** status was used to define TRD (STAR*D response status was defined using **QIDS-C**_**16**_ data, while RIS-INT-93 response status was defined using HAM-D_17_).
(DOCX)

S2 Fig ROC AUC for TRD as defined by responder status and QIDS-C_16_. ROC curves in the training and test dataset (STAR*D) using full set of features, top n features and the overlapping features in all three datasets. (A-C STAR*D training data; D-F STAR*D test data; G RIS-INT-93 test data) where **response** status was used to define TRD (STAR*D response status was defined using **QIDS-C**_**16**_ data, and RIS-INT-93 response status was defined using HAM-D_17_).
(DOCX)

S3 Fig ROC AUC for TRD as defined by remission status and QIDS-SR_16_. ROC curves in the training and test dataset (STAR*D) using full set of features, top n features and the overlapping features in all three datasets. (A-C STAR*D training data; D-F STAR*D test data; G RIS-INT-93 test data) where remission status was used to define TRD (STAR*D remission status was defined using QIDS-SR_16_ data, and RIS-INT-93 remission status was defined using HAM-D_17_).
(DOCX)

S4 Fig ROC AUC for TRD as defined by responder status and QIDS-SR_16_. ROC curves in the training and test dataset (STAR*D) using full set of features, top n features and the overlapping features in all three datasets. (A-C STAR*D training data; D-F STAR*D test data; G RIS-INT-93 test data) where **response** status was used to define TRD (STAR*D response status was defined using **QIDS-SR**_**16**_ data, and RIS-INT-93 response status was defined using HAM-D_17_).
(DOCX)

S5 Fig Variables of importance for outcomes defined using QIDS-SR_16_. Feature importance for outcomes defined by (A) remission status (B) responder status. In both cases, the outcomes were defined using QIDS-SR_**16**_.
(DOCX)

S1 Table Sample size for the training and the testing datasets from STAR*D and an independent RIS-INT-93 dataset. (DOCX)

S2 Table Model performance (outcome defined by QIDS-C_16_). (DOCX)

S3 Table Model performance (outcome defined by QIDS-SR_16_). (DOCX)

S4 Table Confusion matrix for GBDT model in STAR*D test dataset (outcome defined by QIDS-C16) using (a) The 700+ variable setting (b) Top n variable setting (n = 30) (c) The overlapping variable setting and in RIS-INT-93 dataset using (d) The overlapping variable setting.
(DOCX)

S5 Table Model performance using ELNET features selected from the elastic net for the top n (n ~ 30) model. (DOCX)

S6 Table Top 20 groups of predictor variables clustered using k-mean clustering (k = 75) for the outcome defined by remission status using QIDS-C_16_. (DOCX)

S7 Table Predictors from PROC LOGISTIC for TRD phenotype defined using responder criteria defined using QIDS-C_16_. (DOCX)

S8 Table ELNET features and beta coefficient. (DOCX)

S9 Table An overly simplified early response model in the STAR*D training and testing datasets and RIS-INT-93. (DOCX)

## References

[1] Berlim MT, Turecki G. What is the meaning of treatment resistant/refractory major depression (TRD)? A systematic review of current randomized trials. European neuropsychopharmacology: the journal of the European College of Neuropsychopharmacology. 2007;17(11):696–707. doi: 10.1016/j.euroneuro.2007.03.009 . 17521891 10.1016/j.euroneuro.2007.03.009

[2] Ruhe HG, van Rooijen G, Spijker J, Peeters FP, Schene AH. Staging methods for treatment resistant depression. A systematic review. Journal of affective disorders. 2012;137(1–3):35–45. doi: 10.1016/j.jad.2011.02.020 . 21435727 10.1016/j.jad.2011.02.020

[3] Peeters FP, Ruhe HG, Wichers M, Abidi L, Kaub K, van der Lande HJ, et al The Dutch Measure for quantification of Treatment Resistance in Depression (DM-TRD): an extension of the Maudsley Staging Method. Journal of affective disorders. 2016;205:365–71. doi: 10.1016/j.jad.2016.08.019 . 27568174 10.1016/j.jad.2016.08.019

[4] Rush AJ, Trivedi MH, Wisniewski SR, Nierenberg AA, Stewart JW, Warden D, et al Acute and longer-term outcomes in depressed outpatients requiring one or several treatment steps: a STAR*D report. The American journal of psychiatry. 2006;163(11):1905–17. doi: 10.1176/ajp.2006.163.11.1905 . 17074942 10.1176/ajp.2006.163.11.1905

[5] Bennabi D, Aouizerate B, El-Hage W, Doumy O, Moliere F, Courtet P, et al Risk factors for treatment resistance in unipolar depression: A systematic review. Journal of affective disorders. 2014;171C:137–41. doi: 10.1016/j.jad.2014.09.020 . 25305428 10.1016/j.jad.2014.09.020

[6] Souery D, Oswald P, Massat I, Bailer U, Bollen J, Demyttenaere K, et al Clinical factors associated with treatment resistance in major depressive disorder: results from a European multicenter study. The Journal of clinical psychiatry. 2007;68(7):1062–70. . 17685743 10.4088/jcp.v68n0713

[7] Dudek D, Rybakowski JK, Siwek M, Pawlowski T, Lojko D, Roczen R, et al Risk factors of treatment resistance in major depression: association with bipolarity. Journal of affective disorders. 2010;126(1–2):268–71. doi: 10.1016/j.jad.2010.03.001 . 20381154 10.1016/j.jad.2010.03.001

[8] Takahashi M, Shirayama Y, Muneoka K, Suzuki M, Sato K, Hashimoto K. Personality traits as risk factors for treatment-resistant depression. PloS one. 2013;8(5):e63756 doi: 10.1371/journal.pone.0063756 ; PubMed Central PMCID: PMC3661718. 23717477 10.1371/journal.pone.0063756 PMC3661718

[9] Takahashi M, Shirayama Y, Muneoka K, Suzuki M, Sato K, Hashimoto K. Low openness on the revised NEO personality inventory as a risk factor for treatment-resistant depression. PloS one. 2013;8(9):e71964 doi: 10.1371/journal.pone.0071964 ; PubMed Central PMCID: PMC3760870. 24019864 10.1371/journal.pone.0071964 PMC3760870

[10] Sharma V, Khan M, Smith A. A closer look at treatment resistant depression: is it due to a bipolar diathesis? Journal of affective disorders. 2005;84(2–3):251–7. doi: 10.1016/j.jad.2004.01.015 . 15708423 10.1016/j.jad.2004.01.015

[11] Chekroud AM, Zotti RJ, Shehzad Z, Gueorguieva R, Johnson MK, Trivedi MH, et al Cross-trial prediction of treatment outcome in depression: a machine learning approach. The lancet Psychiatry. 2016;3(3):243–50. doi: 10.1016/S2215-0366(15)00471-X . 26803397 10.1016/S2215-0366(15)00471-X

[12] Dodd S, Berk M, Kelin K, Zhang Q, Eriksson E, Deberdt W, et al Application of the Gradient Boosted method in randomised clinical trials: Participant variables that contribute to depression treatment efficacy of duloxetine, SSRIs or placebo. Journal of affective disorders. 2014;168:284–93. doi: 10.1016/j.jad.2014.05.014 . 25080392 10.1016/j.jad.2014.05.014

[13] Kuk AY, Li J, Rush AJ. Recursive subsetting to identify patients in the STAR*D: a method to enhance the accuracy of early prediction of treatment outcome and to inform personalized care. The Journal of clinical psychiatry. 2010;71(11):1502–8. doi: 10.4088/JCP.10m06168blu . 21114950 10.4088/JCP.10m06168blu

[14] Chekroud AM, Gueorguieva R, Krumholz HM, Trivedi MH, Krystal JH, McCarthy G. Reevaluating the Efficacy and Predictability of Antidepressant Treatments: A Symptom Clustering Approach. JAMA psychiatry. 2017;74(4):370–8. doi: 10.1001/jamapsychiatry.2017.0025 . 28241180 10.1001/jamapsychiatry.2017.0025 PMC5863470

[15] Perlis RH. A clinical risk stratification tool for predicting treatment resistance in major depressive disorder. Biological psychiatry. 2013;74(1):7–14. doi: 10.1016/j.biopsych.2012.12.007 ; PubMed Central PMCID: PMC3690142. 23380715 10.1016/j.biopsych.2012.12.007 PMC3690142

[16] Fava M, Rush AJ, Trivedi MH, Nierenberg AA, Thase ME, Sackeim HA, et al Background and rationale for the sequenced treatment alternatives to relieve depression (STAR*D) study. The Psychiatric clinics of North America. 2003;26(2):457–94, x. . 12778843 10.1016/s0193-953x(02)00107-7

[17] Rush AJ, Fava M, Wisniewski SR, Lavori PW, Trivedi MH, Sackeim HA, et al Sequenced treatment alternatives to relieve depression (STAR*D): rationale and design. Controlled clinical trials. 2004;25(1):119–42. . 15061154 10.1016/s0197-2456(03)00112-0

[18] Rapaport MH, Gharabawi GM, Canuso CM, Mahmoud RA, Keller MB, Bossie CA, et al Effects of risperidone augmentation in patients with treatment-resistant depression: Results of open-label treatment followed by double-blind continuation. Neuropsychopharmacology: official publication of the American College of Neuropsychopharmacology. 2006;31(11):2505–13. doi: 10.1038/sj.npp.1301113 . 16760927 10.1038/sj.npp.1301113

[19] Hamilton M. A rating scale for depression. Journal of neurology, neurosurgery, and psychiatry. 1960;23:56–62. ; PubMed Central PMCID: PMC495331. 14399272 10.1136/jnnp.23.1.56 PMC495331

[20] Hamilton M. Development of a rating scale for primary depressive illness. The British journal of social and clinical psychology. 1967;6(4):278–96. . 6080235 10.1111/j.2044-8260.1967.tb00530.x

[21] Boessen R, Groenwold RH, Knol MJ, Grobbee DE, Roes KC. Comparing HAMD(17) and HAMD subscales on their ability to differentiate active treatment from placebo in randomized controlled trials. Journal of affective disorders. 2013;145(3):363–9. doi: 10.1016/j.jad.2012.08.026 . 22959683 10.1016/j.jad.2012.08.026

[22] Bech P, Allerup P, Gram LF, Reisby N, Rosenberg R, Jacobsen O, et al The Hamilton depression scale. Evaluation of objectivity using logistic models. Acta psychiatrica Scandinavica. 1981;63(3):290–9. . 7015793 10.1111/j.1600-0447.1981.tb00676.x

[23] Bech P, Gram LF, Dein E, Jacobsen O, Vitger J, Bolwig TG. Quantitative rating of depressive states. Acta psychiatrica Scandinavica. 1975;51(3):161–70. . 1136841 10.1111/j.1600-0447.1975.tb00002.x

[24] Bech P, Fava M, Trivedi MH, Wisniewski SR, Rush AJ. Factor structure and dimensionality of the two depression scales in STAR*D using level 1 datasets. Journal of affective disorders. 2011;132(3):396–400. doi: 10.1016/j.jad.2011.03.011 . 21440308 10.1016/j.jad.2011.03.011

[25] Maier W, Heuser I, Philipp M, Frommberger U, Demuth W. Improving depression severity assessment—II. Content, concurrent and external validity of three observer depression scales. Journal of psychiatric research. 1988;22(1):13–9. . 3397905 10.1016/0022-3956(88)90023-4

[26] Maier W, Philipp M, Heuser I, Schlegel S, Buller R, Wetzel H. Improving depression severity assessment—I. Reliability, internal validity and sensitivity to change of three observer depression scales. Journal of psychiatric research. 1988;22(1):3–12. . 3397908 10.1016/0022-3956(88)90022-2

[27] Maier W, Philipp M. Comparative analysis of observer depression scales. Acta psychiatrica Scandinavica. 1985;72(3):239–45. . 4072721 10.1111/j.1600-0447.1985.tb02601.x

[28] Santen G, Gomeni R, Danhof M, Della Pasqua O. Sensitivity of the individual items of the Hamilton depression rating scale to response and its consequences for the assessment of efficacy. Journal of psychiatric research. 2008;42(12):1000–9. doi: 10.1016/j.jpsychires.2007.11.004 . 18206909 10.1016/j.jpsychires.2007.11.004

[29] Gibbons RD, Clark DC, Kupfer DJ. Exactly what does the Hamilton Depression Rating Scale measure? Journal of psychiatric research. 1993;27(3):259–73. . 8295158 10.1016/0022-3956(93)90037-3

[30] McIntyre RS, Konarski JZ, Mancini DA, Fulton KA, Parikh SV, Grigoriadis S, et al Measuring the severity of depression and remission in primary care: validation of the HAMD-7 scale. CMAJ: Canadian Medical Association journal = journal de l'Association medicale canadienne. 2005;173(11):1327–34. doi: 10.1503/cmaj.050786 ; PubMed Central PMCID: PMC1283499. 16301700 10.1503/cmaj.050786 PMC1283499

[31] Ostergaard SD, Bech P, Trivedi MH, Wisniewski SR, Rush AJ, Fava M. Brief, unidimensional melancholia rating scales are highly sensitive to the effect of citalopram and may have biological validity: implications for the research domain criteria (RDoC). Journal of affective disorders. 2014;163:18–24. doi: 10.1016/j.jad.2014.03.049 . 24836083 10.1016/j.jad.2014.03.049

[32] Bühlmann P, Rütimann P, van de Geer S, Zhang C-H. Correlated variables in regression: clustering and sparse estimation (with discussion). Journal of Statistical Planning and Inference. 2013;143: 1835–71.

[33] Liu H, Setiono R, editors. Chi2: Feature selection and discretization of numeric attributes. tai; 1995: IEEE.

[34] Zou H, Hastie T. Regularization and variable selection via the elastic net. J R Statist Soc B. 2005;67:301–20.

[35] Friedman J, Hastie T, Tibshirani R. Regularization paths for generalized linear models via coordinate descent. J Stat Softw. 2010;33:1–22. 20808728 PMC2929880

[36] Schimek MG, editor Penalized logistic regression in gene expression analysis. Proceedings to 2003 Semiparametric Conference; 2003; Berlin, Germany.

[37] Shen L, Than EC. Dimension Reduction-Based Penalized Logistic Regression for Cancer Classification Using Microarray Data. EEE/ACM Trans Comput Biol Bioinformatics. 2005;2(2):166–75. 10.1109/TCBB.2005.22 17044181

[38] Breiman L. Random Forests. Machine Learning. 2001;45(1):5–32.

[39] Pedregosa F, Varoquaux G, Gramfort A, Michel V, Thirion B, Grisel O, et al Scikit-learn: Machine Learning in Python. The Journal of Machine Learning Research. 2011;12:2825–30.

[40] Nie Z, Yang T, Liu Y, Lin B, Li Q, Narayan VA, et al Melancholic depression prediction by identifying representative features in metabolic and microarray profiles with missing values. Pacific Symposium on Biocomputing 2015 p. 455–66. 25592604 PMC4299923

[41] Westfall PH, Young SS. Resampling-based multiple testing: examples and methods for P-value adjustment New York: Wiley; 1993 xvii, 340 p. p.

[42] Chase HW, Fournier JC, Greenberg T, Almeida JR, Stiffler R, Zevallos CR, et al Accounting for Dynamic Fluctuations across Time when Examining fMRI Test-Retest Reliability: Analysis of a Reward Paradigm in the EMBARC Study. PloS one. 2015;10(5):e0126326 doi: 10.1371/journal.pone.0126326 ; PubMed Central PMCID: PMC4427400. 25961712 10.1371/journal.pone.0126326 PMC4427400

[43] Greenberg T, Chase HW, Almeida JR, Stiffler R, Zevallos CR, Aslam HA, et al Moderation of the Relationship Between Reward Expectancy and Prediction Error-Related Ventral Striatal Reactivity by Anhedonia in Unmedicated Major Depressive Disorder: Findings From the EMBARC Study. The American journal of psychiatry. 2015;172(9):881–91. doi: 10.1176/appi.ajp.2015.14050594 . 26183698 10.1176/appi.ajp.2015.14050594 PMC4858169

[44] Phillips ML, Chase HW, Sheline YI, Etkin A, Almeida JR, Deckersbach T, et al Identifying predictors, moderators, and mediators of antidepressant response in major depressive disorder: neuroimaging approaches. The American journal of psychiatry. 2015;172(2):124–38. doi: 10.1176/appi.ajp.2014.14010076 ; PubMed Central PMCID: PMC4464814. 25640931 10.1176/appi.ajp.2014.14010076 PMC4464814

[45] Webb CA, Dillon DG, Pechtel P, Goer FK, Murray L, Huys QJ, et al Neural Correlates of Three Promising Endophenotypes of Depression: Evidence from the EMBARC Study. Neuropsychopharmacology: official publication of the American College of Neuropsychopharmacology. 2016;41(2):454–63. doi: 10.1038/npp.2015.165 . 26068725 10.1038/npp.2015.165 PMC5130121

[46] Kennedy SH, Downar J, Evans KR, Feilotter H, Lam RW, MacQueen GM, et al The Canadian Biomarker Integration Network in Depression (CAN-BIND): advances in response prediction. Current pharmaceutical design. 2012;18(36):5976–89. . 22681173 10.2174/138161212803523635

