# Supplementary material for: Predictive modeling of treatment resistant depression using data from STAR*D and an independent clinical study
Source: PLoS One. 2018 Jun 7;13(6):e0197268. doi: 10.1371/journal.pone.0197268 (PMC5991746; doi:10.1371/journal.pone.0197268)
Supplement: S2 Fig — ROC curves in the training and test dataset (STAR*D) using full set of features, top n features and the overlapping features in all three datasets. (A-C STAR*D training data; D-F STAR*D test data; G RIS-INT-93 test data) where response status was used to define TRD (STAR*D response status was defined using QIDS-C16 data, and RIS-INT-93 response status was defined using HAM-D17). (DOCX) [file pone.0197268.s002.docx]

Predictive Modeling of Treatment Resistant Depression using data from STAR*D and an Independent Clinical Study

Zhi Nie^1,2^, Srinivasan Vairavan^3,4^, Vaihbav A. Narayan^3,4^, Jieping Ye^1,2^, and Qingqin S. Li^3,4,*^

**Supporting Information:**

[**S2**](#OLE_LINK4) **Fig** ROC AUC for TRD as defined by **responder** status and **QIDS-C_16_.** ROC curves in the training and test dataset (STAR*D) using full set of features, top n features and the overlapping features in all three datasets. (A-C STAR*D training data; D-F STAR*D test data; G RIS-INT-93 test data) where **response** status was used to define TRD (STAR*D response status was defined using **QIDS-C_16_** data, and RIS-INT-93 response status was defined using HAM-D_17_)

**
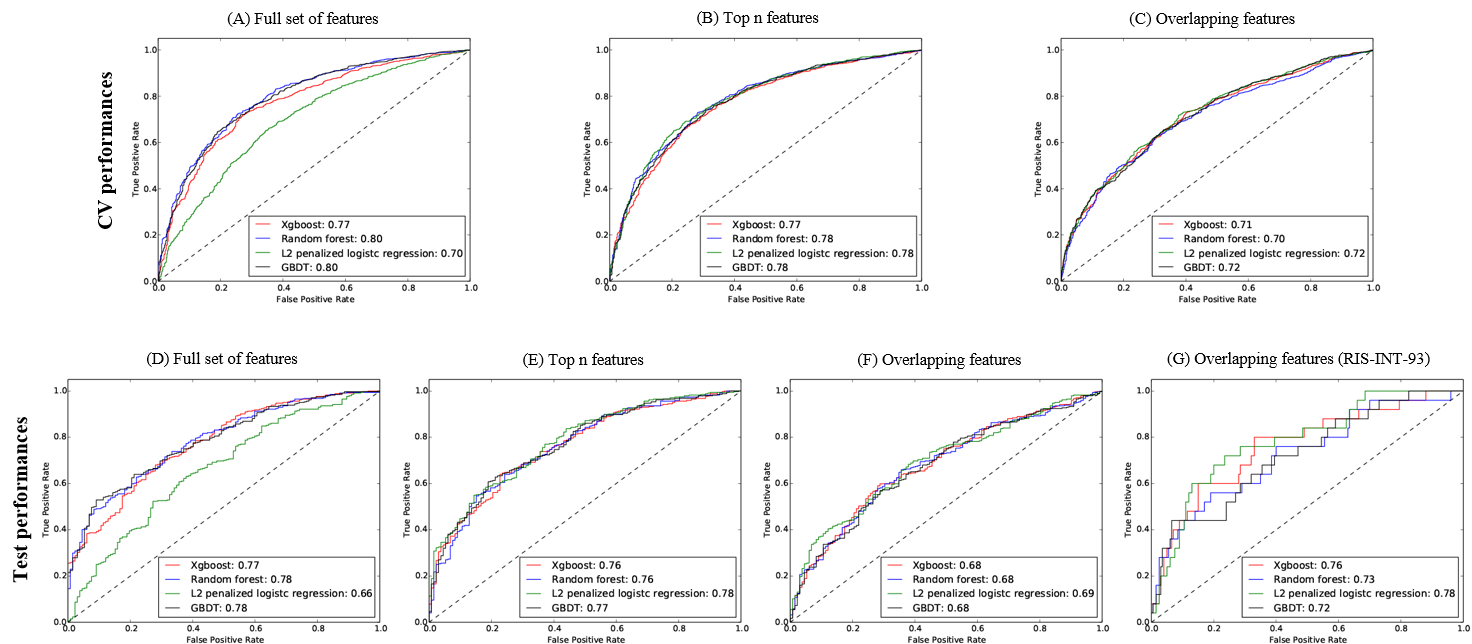
**
