# Supplementary material for: Predictive modeling of treatment resistant depression using data from STAR*D and an independent clinical study
Source: PLoS One. 2018 Jun 7;13(6):e0197268. doi: 10.1371/journal.pone.0197268 (PMC5991746; doi:10.1371/journal.pone.0197268)

Predictive Modeling of Treatment Resistant Depression using data from STAR*D and an Independent Clinical Study

Zhi Nie^1,2^, Srinivasan Vairavan^3,4^, Vaihbav A. Narayan^3,4^, Jieping Ye^1,2^, and Qingqin S. Li^3,4,*^

**Supporting Information:**

[**S3**](#OLE_LINK5) **Fig** ROC AUC for TRD as defined by **remission** status and **QIDS-SR_16._** ROC curves in the training and test dataset (STAR*D) using full set of features, top n features and the overlapping features in all three datasets. (A-C STAR*D training data; D-F STAR*D test data; G RIS-INT-93 test data) where remission status was used to define TRD (STAR*D remission status was defined using QIDS-SR_16_ data, and RIS-INT-93 remission status was defined using HAM-D_17_)


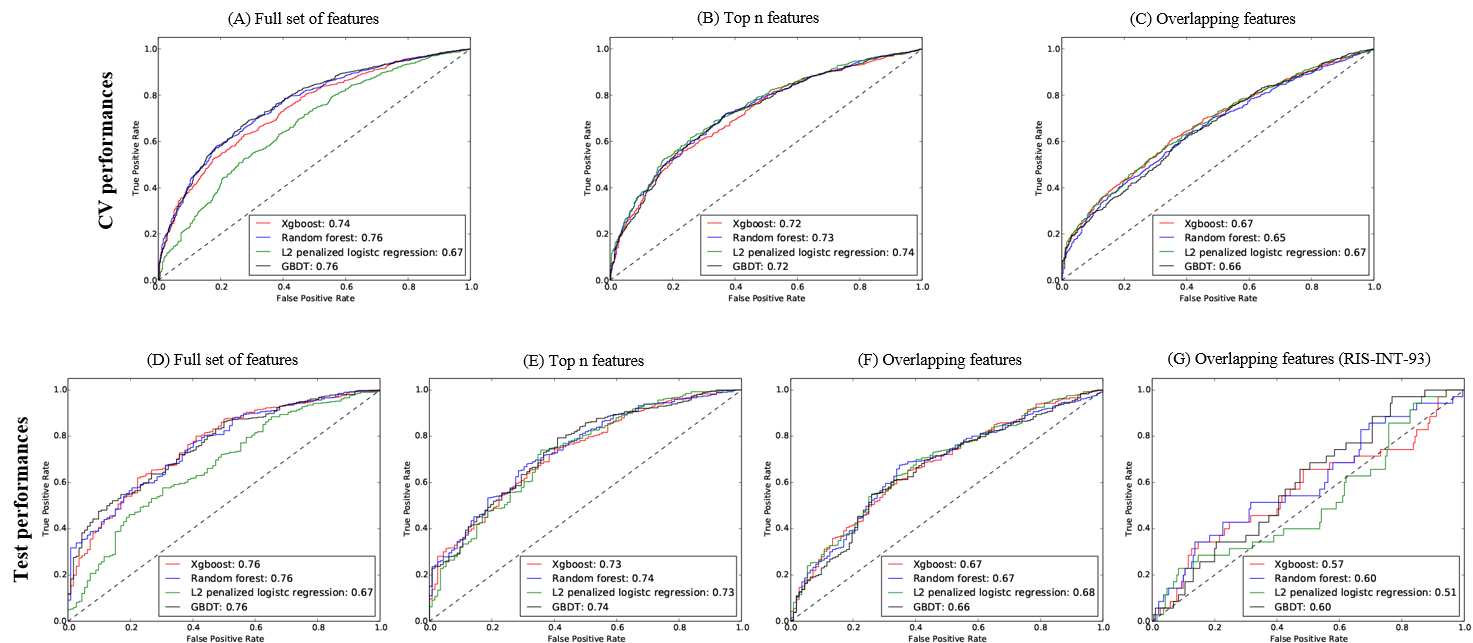

Supplement: S3 Fig — ROC curves in the training and test dataset (STAR*D) using full set of features, top n features and the overlapping features in all three datasets. (A-C STAR*D training data; D-F STAR*D test data; G RIS-INT-93 test data) where remission status was used to define TRD (STAR*D remission status was defined using QIDS-SR16 data, and RIS-INT-93 remission status was defined using HAM-D17). (DOCX) [file pone.0197268.s003.docx]
