# Supplementary material for: Predictive modeling of treatment resistant depression using data from STAR*D and an independent clinical study
Source: PLoS One. 2018 Jun 7;13(6):e0197268. doi: 10.1371/journal.pone.0197268 (PMC5991746; doi:10.1371/journal.pone.0197268)
Supplement: S5 Fig — Feature importance for outcomes defined by (A) remission status (B) responder status. In both cases, the outcomes were defined using QIDS-SR16. (DOCX) [file pone.0197268.s005.docx]

Predictive Modeling of Treatment Resistant Depression using data from STAR*D and an Independent Clinical Study

Zhi Nie^1,2^, Srinivasan Vairavan^3,4^, Vaihbav A. Narayan^3,4^, Jieping Ye^1,2^, and Qingqin S. Li^3,4,*^

**Supporting Information:**

[**S5**](#OLE_LINK7) **Fig** Variables of importance for outcomes defined using **QIDS-SR_16._** Feature importance for outcomes defined by (A) remission status (B) responder status. In both cases, the outcomes were defined using QIDS-SR**_16_**.

(A)

(B)

(B) χ^2^ score
