# Supplementary material for: Predictive modeling of treatment resistant depression using data from STAR*D and an independent clinical study
Source: PLoS One. 2018 Jun 7;13(6):e0197268. doi: 10.1371/journal.pone.0197268 (PMC5991746; doi:10.1371/journal.pone.0197268)
Supplement: S1 Table — (DOCX) [file pone.0197268.s006.docx]

Predictive Modeling of Treatment Resistant Depression using data from STAR*D and an Independent Clinical Study

Zhi Nie^1,2^, Srinivasan Vairavan^3,4^, Vaihbav A. Narayan^3,4^, Jieping Ye^1,2^, and Qingqin S. Li^3,4,*^

**Supporting Information:**

[**S1**](#OLE_LINK10) **Table** Sample size for the training and the testing datasets from STAR*D and an independent RIS-INT-93 dataset

|  | STAR*D Training | STAR*D Testing | RIS-INT-93 |
| --- | --- | --- | --- |
| Regional Centers | 1-8, 10-12 | 13-15 | N/A |
| outcome defined by remission status | | | |
| Sample Size - TRD | 501 | 141 | 200 |
| Sample Size - Non-TRD | 1463 | 349 | 25 |
| outcome defined by responder status | | | |
| Sample Size - TRD | 411 | 104 | 190 |
| Sample Size - Non-TRD | 1797 | 470 | 35 |

Note that the outcome for STAR*D was defined using QIDS-C_16_, while the outcome for RIS-INT-93 was defined using HAM-D_17_
