# Supplementary material for: Predictive modeling of treatment resistant depression using data from STAR*D and an independent clinical study
Source: PLoS One. 2018 Jun 7;13(6):e0197268. doi: 10.1371/journal.pone.0197268 (PMC5991746; doi:10.1371/journal.pone.0197268)
Supplement: S3 Table — (DOCX) [file pone.0197268.s008.docx]

Predictive Modeling of Treatment Resistant Depression using data from STAR*D and an Independent Clinical Study

Zhi Nie^1,2^, Srinivasan Vairavan^3,4^, Vaihbav A. Narayan^3,4^, Jieping Ye^1,2^, and Qingqin S. Li^3,4,*^

**Supporting Information:**

[S3](#Table_S3) Table Model performance (outcome defined by QIDS-SR_16_)

| **Dataset** | **XGBoost** | | | **Random Forest** | | | $l_{2}$ **penalized logistic regression** | | | **GBDT** | | |
| --- | --- | --- | --- | --- | --- | --- | --- | --- | --- | --- | --- | --- |
|  | acc | sst | spc | acc | sst | spc | acc | sst | spc | acc | sst | spc |
| ***Full set of features*** |  |  |  |  |  |  |  |  |  |  |  |  |
| STAR*D Training Dataset |  |  |  |  |  |  |  |  |  |  |  |  |
| outcome defined by remission status | 0.69 | 0.76 | 0.67 | 0.73 | 0.75 | 0.71 | 0.64 | 0.66 | 0.64 | 0.72 | 0.74 | 0.71 |
| outcome defined by response status | 0.63 | 0.75 | 0.60 | 0.67 | 0.72 | 0.66 | 0.61 | 0.62 | 0.61 | 0.68 | 0.72 | 0.67 |
| STAR*D Testing dataset |  |  |  |  |  |  |  |  |  |  |  |  |
| outcome defined by remission status | 0.69 | 0.70 | 0.68 | 0.71 | 0.69 | 0.71 | 0.62 | 0.64 | 0.61 | 0.71 | 0.67 | 0.73 |
| outcome defined by response status | 0.66 | 0.75 | 0.64 | 0.68 | 0.69 | 0.68 | 0.60 | 0.66 | 0.59 | 0.68 | 0.67 | 0.68 |
| ***Top n features (n=30)**** |  |  |  |  |  |  |  |  |  |  |  |  |
| STAR*D Training Dataset |  |  |  |  |  |  |  |  |  |  |  |  |
| outcome defined by remission status | 0.70 | 0.73 | 0.68 | 0.72 | 0.73 | 0.71 | 0.72 | 0.71 | 0.73 | 0.70 | 0.73 | 0.69 |
| outcome defined by response status | 0.62 | 0.71 | 0.60 | 0.64 | 0.69 | 0.63 | 0.68 | 0.67 | 0.68 | 0.64 | 0.71 | 0.63 |
| STAR*D Testing dataset |  |  |  |  |  |  |  |  |  |  |  |  |
| outcome defined by remission status | 0.69 | 0.66 | 0.70 | 0.70 | 0.64 | 0.72 | 0.72 | 0.63 | 0.76 | 0.69 | 0.65 | 0.71 |
| outcome defined by response status | 0.65 | 0.69 | 0.64 | 0.68 | 0.68 | 0.68 | 0.71 | 0.65 | 0.72 | 0.66 | 0.65 | 0.67 |
|  |  |  |  |  |  |  |  |  |  |  |  |  |
| ***Overlapping features*** |  |  |  |  |  |  |  |  |  |  |  |  |
| STAR*D Training Dataset |  |  |  |  |  |  |  |  |  |  |  |  |
| outcome defined by remission status | 0.65 | 0.67 | 0.64 | 0.65 | 0.67 | 0.64 | 0.66 | 0.66 | 0.66 | 0.65 | 0.70 | 0.62 |
| outcome defined by response status | 0.59 | 0.67 | 0.57 | 0.60 | 0.64 | 0.59 | 0.61 | 0.62 | 0.61 | 0.58 | 0.65 | 0.56 |
| STAR*D Testing dataset |  |  |  |  |  |  |  |  |  |  |  |  |
| outcome defined by remission status | 0.64 | 0.59 | 0.65 | 0.63 | 0.65 | 0.63 | 0.65 | 0.63 | 0.66 | 0.63 | 0.62 | 0.64 |
| outcome defined by response status | 0.62 | 0.64 | 0.61 | 0.61 | 0.70 | 0.59 | 0.66 | 0.62 | 0.67 | 0.61 | 0.68 | 0.59 |
| RIS-INT-93 dataset |  |  |  |  |  |  |  |  |  |  |  |  |
| outcome defined by response status | 0.85 | 0.91 | 0.40 | 0.87 | 0.94 | 0.36 | 0.84 | 0.90 | 0.40 | 0.87 | 0.93 | 0.44 |
| outcome defined by response status | 0.84 | 1.00 | 0.00 | 0.84 | 0.99 | 0.03 | 0.84 | 1.00 | 0.00 | 0.84 | 0.99 | 0.03 |

acc: accuracy; spc: specificity; sst: sensitivity

*features selected using clustering-$\chi^{2}$
