# Supplementary material for: Predictive modeling of treatment resistant depression using data from STAR*D and an independent clinical study
Source: PLoS One. 2018 Jun 7;13(6):e0197268. doi: 10.1371/journal.pone.0197268 (PMC5991746; doi:10.1371/journal.pone.0197268)
Supplement: S4 Table — Confusion matrix for GBDT model in STAR*D test dataset (outcome defined by QIDS-C16) using (a) The 700+ variable setting (b) Top n variable setting (n = 30) (c) The overlapping variable setting and in RIS-INT-93 dataset using (d) The overlapping variable setting. (DOCX) [file pone.0197268.s009.docx]

Predictive Modeling of Treatment Resistant Depression using data from STAR*D and an Independent Clinical Study

Zhi Nie^1,2^, Srinivasan Vairavan^3,4^, Vaihbav A. Narayan^3,4^, Jieping Ye^1,2^, and Qingqin S. Li^3,4,*^

**Supporting Information:**

**S4**  **Table** Confusion matrix for GBDT model in STAR*D test dataset (outcome defined by QIDS-C16) using (a) The 700+ variable setting (b) Top n variable setting (n = 30) (c) The overlapping variable setting and in RIS-INT-93 dataset using (d) The overlapping variable setting

(a)

|  | Ground Truth | |
| --- | --- | --- |
| Predicted | TRD | non-TRD |
| TRD | 97 | 102 |
| non-TRD | 44 | 247 |

(b)

| \|  \| \| --- \| | Ground Truth | |
| --- | --- | --- | --- |
| Predicted | TRD | non-TRD |
| TRD | 102 | 110 |
| non-TRD | 39 | 239 |

(c)

|  | Ground Truth | |
| --- | --- | --- |
| Predicted | TRD | non-TRD |
| TRD | 99 | 124 |
| non-TRD | 42 | 225 |

(d)

|  | Ground Truth | |
| --- | --- | --- |
| Predicted | TRD | non-TRD |
| TRD | 175 | 15 |
| non-TRD | 25 | 10 |
