# Supplementary material for: Predictive modeling of treatment resistant depression using data from STAR*D and an independent clinical study
Source: PLoS One. 2018 Jun 7;13(6):e0197268. doi: 10.1371/journal.pone.0197268 (PMC5991746; doi:10.1371/journal.pone.0197268)
Supplement: S5 Table — (DOCX) [file pone.0197268.s010.docx]

Predictive Modeling of Treatment Resistant Depression using data from STAR*D and an Independent Clinical Study

Zhi Nie^1,2^, Srinivasan Vairavan^3,4^, Vaihbav A. Narayan^3,4^, Jieping Ye^1,2^, and Qingqin S. Li^3,4,*^

**Supporting Information:**

[**S5**](#Table_S5) **Table** Model performance using ELNET features selected from the elastic net for the top n (n ~ 30) model

| Outcome measurement | acc | spc | sst | auc |
| --- | --- | --- | --- | --- |
|  |  | XGBoost |  |  |
| QIDS-SR_16_/Remission | 0.68 | 0.67 | 0.69 | 0.76 |
| QIDS-C_16_/Remission | 0.67 | 0.65 | 0.72 | 0.76 |
| QIDS-SR_16_/Response | 0.65 | 0.63 | 0.72 | 0.76 |
| QIDS-C_16_/Response | 0.64 | 0.61 | 0.77 | 0.75 |
|  |  | Random forest | |  |
| QIDS-SR_16_/Remission | 0.71 | 0.72 | 0.68 | 0.77 |
| QIDS-C_16_/Remission | 0.70 | 0.70 | 0.68 | 0.76 |
| QIDS-SR_16_/Response | 0.67 | 0.67 | 0.67 | 0.75 |
| QIDS-C_16_/Response | 0.69 | 0.68 | 0.72 | 0.76 |
|  |  | $l_{2}$ penalized logistic regression | | |
| QIDS-SR_16_/Remission | 0.72 | 0.76 | 0.61 | 0.78 |
| QIDS-C_16_/Remission | 0.72 | 0.73 | 0.68 | 0.77 |
| QIDS-SR_16_/Response | 0.71 | 0.73 | 0.66 | 0.74 |
| QIDS-C_16_/Response | 0.69 | 0.69 | 0.70 | 0.77 |
|  |  | GBDT |  |  |
| QIDS-SR_16_/Remission | 0.73 | 0.74 | 0.69 | 0.78 |
| QIDS-C_16_/Remission | 0.70 | 0.71 | 0.68 | 0.76 |
| QIDS-SR_16_/Response | 0.68 | 0.68 | 0.67 | 0.76 |
| QIDS-C_16_/Response | 0.68 | 0.67 | 0.72 | 0.77 |
|  |  | Elastic Net |  |  |
| QIDS-SR_16_/Remission | 0.71 | 0.74 | 0.64 | 0.76 |
| QIDS-C_16_/Remission | 0.70 | 0.73 | 0.64 | 0.76 |
| QIDS-SR_16_/Response | 0.70 | 0.73 | 0.60 | 0.74 |
| QIDS-C_16_/Response | 0.70 | 0.71 | 0.66 | 0.74 |
