# Supplementary material for: Predictive modeling of treatment resistant depression using data from STAR*D and an independent clinical study
Source: PLoS One. 2018 Jun 7;13(6):e0197268. doi: 10.1371/journal.pone.0197268 (PMC5991746; doi:10.1371/journal.pone.0197268)
Supplement: S6 Table — (DOCX) [file pone.0197268.s011.docx]

Predictive Modeling of Treatment Resistant Depression using data from STAR*D and an Independent Clinical Study

Zhi Nie^1,2^, Srinivasan Vairavan^3,4^, Vaihbav A. Narayan^3,4^, Jieping Ye^1,2^, and Qingqin S. Li^3,4,*^

**Supporting Information:**

[**S6**](#Table_S6) **Table** Top 20 groups of predictor variables clustered using k-mean clustering (k = 75) for the outcome defined by **remission** status using **QIDS-C_16_** (please refer to STAR*D data dictionary for variable description)

| 1 |  |
| --- | --- |
|  | L1_W_2_CC_QCCUR_R |
|  | L1_W_2_CC_QSCUR_R |
|  | L1_W_2_CC_CGI_I |
|  | L1_W_2_CC_RAISE |
|  | L1_W_2_QC_CMDSD |
|  | L1_W_2_QC_CCNTR |
|  | L1_W_2_QC_CINTR |
|  | L1_W_2_QC_CENGY |
|  | L1_W_2_QC_CSLOW |
|  | L1_W_2_QC_QCTOT_R |
|  | L1_W_2_QS_SMDSD |
|  | L1_W_2_QS_SCNTR |
|  | L1_W_2_QS_SINTR |
|  | L1_W_2_QS_SENGY |
|  | L1_W_2_QS_SSLOW |
|  | L1_W_2_QS_QSTOT |
|  | L1_W_2_QC_CMDSD_ratio |
|  | L1_W_2_QC_CCNTR_ratio |
|  | L1_W_2_QC_CINTR_ratio |
|  | L1_W_2_QC_CENGY_ratio |
|  | L1_W_2_QC_CSLOW_ratio |
|  | L1_W_2_QC_QCTOT_R_ratio |
|  | L1_W_2_QS_SMDSD_ratio |
|  | L1_W_2_QS_SCNTR_ratio |
|  | L1_W_2_QS_SINTR_ratio |
|  | L1_W_2_QS_SENGY_ratio |
|  | L1_W_2_QS_SSLOW_ratio |
|  | L1_W_2_QS_QSTOT_ratio |
|  | PCHG2wk |
|  | mPCHG2wk |
| 2 |  |
|  | L1_W_0_IVR_assmt_SFHS09 |
|  | L1_W_0_IVR_assmt_SFHS10 |
|  | L1_W_0_IVR_assmt_SFHS11 |
|  | L1_W_0_IVR_assmt_SFHS12 |
|  | L1_W_0_IVR_assmt_MCS12 |
|  | L1_W_0_IVR_assmt_QLESQ02 |
|  | L1_W_0_IVR_assmt_QLESQ03 |
|  | L1_W_0_IVR_assmt_QLESQ04 |
|  | L1_W_0_IVR_assmt_QLESQ05 |
|  | L1_W_0_IVR_assmt_QLESQ06 |
|  | L1_W_0_IVR_assmt_QLESQ07 |
|  | L1_W_0_IVR_assmt_QLESQ08 |
|  | L1_W_0_IVR_assmt_QLESQ09 |
|  | L1_W_0_IVR_assmt_QLESQ10 |
|  | L1_W_0_IVR_assmt_QLESQ11 |
|  | L1_W_0_IVR_assmt_QLESQ14 |
|  | L1_W_0_IVR_assmt_QLESQ15 |
|  | L1_W_0_IVR_assmt_QLESQ16 |
|  | L1_W_0_IVR_assmt_totQLESQ |
| 3 |  |
|  | L1_W_0_IVR_assmt_VINTR |
|  | L1_W_0_IVR_assmt_VENGY |
|  | L1_W_0_IVR_assmt_QVTOT |
|  | L1_W_0_IVR_assmt_WPAI05 |
|  | L1_W_0_IVR_assmt_WPAI06 |
|  | L1_W_0_IVR_assmt_WPAI_PctWrkImp |
|  | L1_W_0_IVR_assmt_WPAI_PctActImp |
|  | L1_W_0_IVR_assmt_WPAI_TotWrkImp |
|  | L1_W_0_IVR_assmt_WSAS01 |
|  | L1_W_0_IVR_assmt_WSAS02 |
|  | L1_W_0_IVR_assmt_WSAS03 |
|  | L1_W_0_IVR_assmt_WSAS04 |
|  | L1_W_0_IVR_assmt_WSAS05 |
|  | L1_W_0_IVR_assmt_totWSAS |
|  | L1_W_0_IVR_assmt_WSAStot |
| 4 |  |
|  | L1_W_2_QC_CSUIC |
|  | L1_W_2_QS_SSUIC |
|  | L1_W_2_QC_CSUIC_ratio |
|  | L1_W_2_QS_SSUIC_ratio |
| 5 |  |
|  | L1_W_0_IVR_assmt_SFHS01 |
|  | L1_W_0_IVR_assmt_SFHS02 |
|  | L1_W_0_IVR_assmt_SFHS03 |
|  | L1_W_0_IVR_assmt_SFHS04 |
|  | L1_W_0_IVR_assmt_SFHS05 |
|  | L1_W_0_IVR_assmt_SFHS08 |
|  | L1_W_0_IVR_assmt_PCS12 |
|  | L1_W_0_IVR_assmt_QLESQ01 |
|  | L1_W_0_IVR_assmt_QLESQ12 |
|  | L1_W_0_IVR_assmt_QLESQ13 |
| 6 |  |
|  | L1_W_2_QC_CVWSF |
|  | L1_W_2_QS_SVWSF |
|  | L1_W_2_QC_CVWSF_ratio |
|  | L1_W_2_QS_SVWSF_ratio |
| 7 |  |
|  | HRSD_HINTR |
|  | HRSD_HENGY |
|  | HRSD_HDTOT_R |
|  | PDS_JOY2W |
|  | PDS_INT2W |
|  | PDS_TRD2W |
|  | L1_W_0_QC_CINTR |
|  | L1_W_0_QC_CENGY |
|  | L1_W_0_QC_QCTOT_R |
|  | L1_W_0_QS_SINTR |
|  | L1_W_0_QS_SENGY |
|  | L1_W_0_QS_QSTOT |
|  | L1_SC_HRSD_R |
|  | L1_SC_QCCUR_R |
|  | L1_SC_QSCUR_R |
|  | L1_W_2_CC_QCBEG_R |
|  | C_IDSC5 |
| 8 |  |
|  | L1_W_0_RA_HMDSD |
|  | L1_W_0_RA_HINTR |
|  | L1_W_0_RA_HENGY |
|  | L1_W_0_RA_IMDSD |
|  | L1_W_0_RA_IRCT |
|  | L1_W_0_RA_IINTR |
|  | L1_W_0_RA_IPLSR |
|  | L1_W_0_RA_IENGY |
|  | Bech |
|  | Maier |
|  | Gibbons |
|  | Santen |
|  | McIntyre |
|  | F_RET |
| 9 |  |
|  | L1_W_0_RA_HSANX |
|  | L1_W_0_RA_HHYPC |
|  | L1_W_0_RA_ISMTC |
|  | L1_W_0_RA_ISYMP |
|  | L1_W_0_RA_ILDN |
|  | F_ANX |
| 10 |  |
|  | L1_W_2_CC_ADMD1=103 |
|  | L1_W_2_CC_STMED |
|  | L1_W_2_CC_STMD1=103 |
|  | L1_W_2_CC_STMD2=103 |
|  | L1_W_2_CC_ADDS1=103 |
|  | L1_W_2_CC_STDS1=103 |
|  | L1_W_2_CC_STDS2=103 |
| 11 |  |
|  | PDS_ANAVD |
|  | PDS_FRAVD |
|  | PDS_FRFAR |
|  | PDS_FRCWD |
|  | PDS_FRLNE |
|  | PDS_FRBRG |
|  | PDS_FRBUS |
|  | PDS_FRCAR |
|  | PDS_FRALO |
|  | PDS_FROPN |
|  | PDS_FRANX |
|  | PDS_FRSIT |
| 12 |  |
|  | L1_W_2_PRS_GDIAR |
|  | L1_W_2_PRS_GDMTH |
|  | L1_W_2_PRS_GNSEA |
|  | L1_W_2_PRS_GSTRO |
|  | L1_W_2_PRS_HTCHS |
|  | L1_W_2_PRS_NVHED |
|  | L1_W_2_PRS_NVTRM |
|  | L1_W_2_PRS_NVCRD |
|  | L1_W_2_PRS_NRVSY |
|  | L1_W_2_PRS_EYVSN |
|  | L1_W_2_PRS_EARNG |
|  | L1_W_2_PRS_EYEAR |
|  | L1_W_2_PRS_URFRQ |
|  | L1_W_2_PRS_GENUR_PRS |
|  | L1_W_2_PRS_SLDIF |
|  | L1_W_2_PRS_SLEEP |
|  | L1_W_2_PRS_OAXTY |
|  | L1_W_2_PRS_OCTRT |
|  | L1_W_2_PRS_OMAL |
|  | L1_W_2_PRS_OFTGE |
|  | L1_W_2_PRS_ODEGY |
|  | L1_W_2_PRS_OTHER_PRS |
| 13 |  |
|  | L1_W_2_CC_STMD1=101 |
|  | L1_W_2_CC_STMD2=101 |
|  | L1_W_2_CC_TRTMT |
|  | L1_W_2_CC_STDS1=101 |
|  | L1_W_2_CC_STDS2=101 |
| 14 |  |
|  | HRSD_HSOIN |
|  | HRSD_HMNIN |
|  | HRSD_HEMIN |
|  | EL_DSMIH |
|  | PDS_LSL2W |
|  | L1_W_0_QC_CSOIN |
|  | L1_W_0_QC_CMNIN |
|  | L1_W_0_QC_CEMIN |
|  | L1_W_0_QS_SSOIN |
|  | L1_W_0_QS_SMNIN |
|  | L1_W_0_QS_SEMIN |
|  | L1_W_0_IVR_assmt_VSOIN |
|  | L1_W_0_IVR_assmt_VMNIN |
|  | L1_W_0_IVR_assmt_VEMIN |
|  | L1_W_0_IVR_assmt_VQC1_4 |
|  | L1_W_0_RA_HSOIN |
|  | L1_W_0_RA_HMNIN |
|  | L1_W_0_RA_HEMIN |
|  | L1_W_0_RA_ISOIN |
|  | L1_W_0_RA_IMNIN |
|  | L1_W_0_RA_IEMIN |
|  | L1_W_2_QC_CSOIN |
|  | L1_W_2_QC_CMNIN |
|  | L1_W_2_QC_CEMIN |
|  | L1_W_2_QS_SSOIN |
|  | L1_W_2_QS_SMNIN |
|  | L1_W_2_QS_SEMIN |
|  | F_SLE |
| 15 |  |
|  | L1_W_2_CC_STMD1=107 |
|  | L1_W_2_CC_STDS1=107 |
|  | L1_W_2_QS_SUPPTHRP |
|  | L1_W_2_QS_SUPPTHRP_ratio |
| 16 |  |
|  | HRSD_HSLOW |
|  | EL_DSMDT |
|  | PDS_CNT2W |
|  | PDS_DCN2W |
|  | L1_W_0_QC_CCNTR |
|  | L1_W_0_QC_CSLOW |
|  | L1_W_0_QS_SCNTR |
|  | L1_W_0_QS_SSLOW |
|  | L1_W_0_IVR_assmt_VCNTR |
|  | L1_W_0_IVR_assmt_VSLOW |
|  | L1_W_0_RA_HSLOW |
|  | L1_W_0_RA_ICNTR |
|  | L1_W_0_RA_ISLOW |
| 17 |  |
|  | HRSD_HSUIC |
|  | EL_DSMTD |
|  | PDS_PSV2W |
|  | PDS_WSH2W |
|  | PDS_BTR2W |
|  | PDS_THT2W |
|  | PDS_SER2W |
|  | PDS_SPF2W |
|  | L1_W_0_QC_CSUIC |
|  | L1_W_0_QS_SSUIC |
|  | L1_W_0_IVR_assmt_VSUIC |
|  | L1_W_0_RA_HSUIC |
|  | L1_W_0_RA_ISUIC |
| 18 |  |
|  | DM_SCHOOL |
|  | DM_DEGREE |
| 19 |  |
|  | L1_W_2_PRS_HTPLP |
|  | L1_W_2_PRS_HTDZY |
|  | L1_W_2_PRS_HEART_PRS |
|  | L1_W_2_PRS_NVDZY |
| 20 |  |
|  | L1_W_2_CC_QCIMP_R |
|  | L1_W_2_CC_REMSN |
|  | L1_W_2_PRS_GNONE |
|  | L1_W_2_PRS_HTNONE |
|  | L1_W_2_PRS_SKNONE |
|  | L1_W_2_PRS_NVNONE |
|  | L1_W_2_PRS_ENONE |
|  | L1_W_2_PRS_URNONE |
|  | L1_W_2_PRS_SLNONE |
|  | L1_W_2_PRS_SXNONE |
|  | L1_W_2_PRS_ONONE |
