# Supplementary material for: Predictive modeling of treatment resistant depression using data from STAR*D and an independent clinical study
Source: PLoS One. 2018 Jun 7;13(6):e0197268. doi: 10.1371/journal.pone.0197268 (PMC5991746; doi:10.1371/journal.pone.0197268)
Supplement: S7 Table — (DOCX) [file pone.0197268.s012.docx]

Predictive Modeling of Treatment Resistant Depression using data from STAR*D and an Independent Clinical Study

Zhi Nie^1,2^, Srinivasan Vairavan^3,4^, Vaihbav A. Narayan^3,4^, Jieping Ye^1,2^, and Qingqin S. Li^3,4,*^

**Supporting Information:**

[**S7**](#Table_S8) **Table** predictors from PROC LOGISTIC for TRD phenotype defined using **responder** criteria defined using **QIDS-C_16_**

| Effect | Pr > ChiSq | Odds Ratio Point Estimate | Odds Ratio 95% Wald Confidence Limits |
| --- | --- | --- | --- |
| QIDS-C_16_ total score at week 2 (Level 1) | <.0001 | 1.159 | (1.120, 1.200) |
| PRISE: If you had any symptoms (anxiety, poor concentration, general malaise, or restlessness) over the last week, how bad was your worst symptom? (0 - not present, 1- tolerable, 2- distressing) | 0.0309 | 1.220 | (1.018, 1.461) |
| QLESQ total score at week 0 (Level 1) | 0.0283 | 0.987 | (0.975, 0.999) |
| WSAS total score | 0.0283 | 1.023 | (1.003, 1.042) |
| SFHS physical component | 0.0043 | 0.984 | (0.974, 0.995) |
| HAM-D Anxiety Subtotal at week 0 (Level 1) | 0.0137 | 0.931 | (0.880, 0.985) |
| QIDS-SR**_16_** total score at week 0 (Level 1) | 0.0072 | 0.952 | (0.918, 0.987) |
| PRISE: if you had any symptoms (palpitation, dizziness on standing, chest pain) over the last week, how bad was your worst symptom? (0 - not present, 1- tolerable, 2- distressing) | 0.0660 | 1.214 | (0.987, 1.492) |
| PRISE: If you had any symptoms (difficulty urinating, painful urination, frequent urination, menstrual irregularity) over the last week, how bad was your WORST symptom? (0 - not present, 1- tolerable, 2- distressing) | 0.0087 | 1.343 | (1.077, 1.673) |
| PDSQ: Did you obsessively and excessively check or repeat things over and over again? | 0.0086 | 1.458 | (1.100, 1.931) |
| Patient is presently not in remission. Dose will not be raised due to side effects preventing a dose increase. | 0.0267 | 0.884 | (0.792, 0.986) |
| In the course of the current MDE, does the patient regularly experience worsening of depressive symptoms 5-10 days prior to menses? 1 = Yes, 0 = No, -3 = unknown, -2 = not applicable) | 0.0333 | 0.906 | (0.828, 0.992) |
| PDSQ: Did you think about a specific way of taking your life? | 0.0457 | 1.304 | (1.005, 1.693) |

*Sample size for final logistic regression model (411 TRD vs. 1797 non-TRD)
