# Supplementary material for: Predictive modeling of treatment resistant depression using data from STAR*D and an independent clinical study
Source: PLoS One. 2018 Jun 7;13(6):e0197268. doi: 10.1371/journal.pone.0197268 (PMC5991746; doi:10.1371/journal.pone.0197268)
Supplement: S8 Table — (DOCX) [file pone.0197268.s013.docx]

Predictive Modeling of Treatment Resistant Depression using data from STAR*D and an Independent Clinical Study

Zhi Nie^1,2^, Srinivasan Vairavan^3,4^, Vaihbav A. Narayan^3,4^, Jieping Ye^1,2^, and Qingqin S. Li^3,4,*^

**Supporting Information:**

[**S8**](#Table_S7) **Table** ELNET features and β coefficient

| Variable | Source Form* | beta coefficient |
| --- | --- | --- |
| Level 1 QIDS-C_16_ Total score at week 2 | QC | 1.68E+00 |
| Level 1 QIDS-C_16_ Total score at week 2 | CC | 1.40E+00 |
| Level 1 QIDS-C_16_ Mood (sad) item score at week 2 | QC | 8.02E-01 |
| Level 1 QIDS-SR_16_ Total score at week 2 | QS | 7.81E-01 |
| Level 1 QLESQ total score at week 0 | IVR | -6.72E-01 |
| Level 1 QIDS-SR_16_ Mood (sad) item score at week 2 | QS | 5.24E-01 |
| Level 1 WSAS Social activities impaired item score at week 0 | IVR | 3.63E-01 |
| Level 1 CGI-I score at week 2 | CC | 3.17E-01 |
| Level 1 QIDS-C_16_ Total score percent improvement at week 2 | CC | -3.08E-01 |
| Level 1 QIDS-C_16_ Concentration/decision making item score at week 2 | QC | 3.05E-01 |
| Level 1 WSAS total score at week 0 | IVR | 2.96E-01 |
| Level 1 WSAS total score at week 0 | IVR | 2.94E-01 |
| Level 1 SFHS physical component at week 0 | IVR | -2.52E-01 |
| Level 1 QIDS-C_16_ Total score percent improvement at week 2 | derived | 8.67E-02 |
| Level 1 QIDS-C_16_ Total score percent improvement at week 2 | QC | 6.77E-02 |
| Level 1 QIDS-C_16_ Energy/fatigability item score at week 2 | QC | 6.18E-02 |
| Level 1 SFHS Limited in climbing flights of stairs item score at week 0 | IVR | -2.62E-02 |
| Level 1 QLESQ Physical mobility item score at week 0 | IVR | -2.03E-02 |
| Level 1 QIDS Sleep onset insomnia item score at week 0 | IVR | 1.86E-02 |
| Level 1 QIDS total score at week 0 | IVR | 1.69E-02 |
| IDS-C_5_ | derived | 1.30E-02 |
| Level 1 QIDS Highest score 15-16 (Psychomotor agitation/retardation) item score at week 0 | IVR | 8.19E-03 |
| Level 1 QLESQ Vision item score at week 0 | IVR | -7.10E-03 |
| Level 1 QIDS-C_16_ Involvement item score at week 2 | QC | 7.05E-03 |
| Level 1 IDS-C_5_ Total score percent improvement at week 2 | CC | 2.05E-03 |
| Level 1 QIDS-SR_16_ total score at week 2 | CC | 1.24E-03 |
| Level 1 SFHS Pain interfered with normal work item score at week 0 | IVR | -9.29E-04 |
| Musculoskeletal/Integument problem | CRS | 7.43E-04 |
| Level 1 HRS-D current score (transcribed) | SC | 6.60E-04 |
| HRS total score at enrollment | HRSD | 8.89E-05 |
| PH Bothered by aches/pains | PDS | 7.94E-05 |
