# Supplementary material for: Predictive modeling of treatment resistant depression using data from STAR*D and an independent clinical study
Source: PLoS One. 2018 Jun 7;13(6):e0197268. doi: 10.1371/journal.pone.0197268 (PMC5991746; doi:10.1371/journal.pone.0197268)
Supplement: S9 Table — (DOCX) [file pone.0197268.s014.docx]

Predictive Modeling of Treatment Resistant Depression using data from STAR*D and an Independent Clinical Study

Zhi Nie^1,2^, Srinivasan Vairavan^3,4^, Vaihbav A. Narayan^3,4^, Jieping Ye^1,2^, and Qingqin S. Li^3,4,*^

**Supporting Information:**

[**S9**](#OLE_LINK12) **Table** An overly simplified early response model in the STAR*D training and testing datasets and RIS-INT-93

|  | Accuracy | Sensitivity | Specificity | PPV | NPV |
| --- | --- | --- | --- | --- | --- |
| outcome defined by remission status | | | | | |
| STAR*D Training | 0.62 | 0.52 | 0.77 | 0.75 | 0.54 |
| STAR*D testing | 0.60 | 0.49 | 0.76 | 0.74 | 0.52 |
| RIS-INT-93 | 0.66 | 0.65 | 0.76 | 0.96 | 0.21 |
| outcome defined by responder status | | | | | |
| STAR*D Training | 0.67 | 0.58 | 0.75 | 0.64 | 0.69 |
| STAR*D testing | 0.66 | 0.55 | 0.74 | 0.61 | 0.70 |
| RIS-INT-93 | 0.67 | 0.66 | 0.71 | 0.93 | 0.28 |

PPV: positive predictive value; NPV: negative predictive value
